# The Importance of Objective Stool Classification in Fecal 1H-NMR Metabolomics: Exponential Increase in Stool Crosslinking Is Mirrored in Systemic Inflammation and Associated to Fecal Acetate and Methionine

**DOI:** 10.3390/metabo11030172

**Published:** 2021-03-16

**Authors:** Leon Deutsch, Blaz Stres

**Affiliations:** 1 Biotechnical Faculty, University of Ljubljana, Jamnikarjeva 101, SI-1000 Ljubljana, Slovenia; leon.deutsch@bf.uni-lj.si; 2 Faculty of Civil and Geodetic Engineering, University of Ljubljana, Jamova 2, SI-1000 Ljubljana, Slovenia; 3 Department of Automation, Jožef Stefan Institute, Biocybernetics and Robotics, Jamova 39, SI-1000 Ljubljana, Slovenia; 4 Department of Microbiology, University of Innsbruck, Technikerstrasse 25d, A-6020 Innsbruck, Austria

**Keywords:** feces, stool, classification, Bristol stool scale, BSS, JADBIO, gel network, intestinal abrasion

## Abstract

Past studies strongly connected stool consistency—as measured by Bristol Stool Scale (BSS)—with microbial gene richness and intestinal inflammation, colonic transit time and metabolome characteristics that are of clinical relevance in numerous gastro intestinal conditions. While retention time, defecation rate, BSS but not water activity have been shown to account for BSS-associated inflammatory effects, the potential correlation with the strength of a gel in the context of intestinal forces, abrasion, mucus imprinting, fecal pore clogging remains unexplored as a shaping factor for intestinal inflammation and has yet to be determined. Our study introduced a minimal pressure approach (MP) by probe indentation as measure of stool material crosslinking in fecal samples. Results reported here were obtained from 170 samples collected in two independent projects, including males and females, covering a wide span of moisture contents and BSS. MP values increased exponentially with increasing consistency (i.e., lower BSS) and enabled stratification of samples exhibiting mixed BSS classes. A trade-off between lowest MP and highest dry matter content delineated the span of intermediate healthy density of gel crosslinks. The crossectional transects identified fecal surface layers with exceptionally high MP and of <5 mm thickness followed by internal structures with an order of magnitude lower MP, characteristic of healthy stool consistency. The MP and BSS values reported in this study were coupled to reanalysis of the PlanHab data and fecal 1H-NMR metabolomes reported before. The exponential association between stool consistency and MP determined in this study was mirrored in the elevated intestinal and also systemic inflammation and other detrimental physiological deconditioning effects observed in the PlanHab participants reported before. The MP approach described in this study can be used to better understand fecal hardness and its relationships to human health as it provides a simple, fine scale and objective stool classification approach for the characterization of the exact sampling locations in future microbiome and metabolome studies.

## 1. Introduction

The contractile patterns of small intestine propagate toward the colon and are caused by the enteric nervous system (the interstitial cells of Cajal) that generate slow waves of smooth muscle contraction and contribute to the transit rates along the intestine [1,2,3]. The number and the length of peristaltic waves, i.e., circular constrictions propagating aborally, determine chime transport that decreases along the gut in the same proportion as the volume of luminal content declines by absorption of nutrients and water [4,5]. Repeated contractions are essential for maintenance of a steady-state bacterial population as mixing of chime helps to overcome flow, and controlled contractions by the colon strongly influence microbiota density and composition. Consequently, flow and mixing play a major role in shaping the microbial metabolic reactions and interactions with the host [6]. In addition, with increasing filling of distal intestinal segments (i.e., constipation), the motility of the proximal intestine is inhibited, the number of peristaltic waves decreases while the number of stationary segmenting contractions increases [1,2,3]. In addition to peristaltic waves, stationary or segmenting contractions are pushing, mixing and separating chime into segments. These contractions are isolated at a single site without spatio-temporal pattern and are responsible for inflicting intestinal abrasions during prolonged and increasing constipation. The derived intestinal abrasions are highly amenable for further microbial colonization at locations with a thin (e.g., small intestine) or modified mucus layer (e.g., abrasions, imprinting, reduced mucus thickness, increased porosity and modified mucus glycosylation pattern; [7]). Local pH values in the lumen differentially affect the growth of different bacteria and drive changes in microbiota composition. The key factors influencing the delicate regulation of colonic pH are epithelial water absorption, nutrient inflow, and luminal buffering capacity [5].

Stool consistency was strongly associated with the gut microbiota richness and composition, enterotypes, increased local inflammation, lipopolysaccharides and bacterial growth rates [8,9]. Stool consistency is generally assessed using the widely adopted Bristol Stool Scale (BSS) [10,11]. In addition, long colonic transit time corresponded to lower BSS score, higher microbial richness and a shift in colonic metabolism from carbohydrate fermentation towards protein catabolism [12], interlinking thus the status of systemic levels of metabolites with microbiome and BSS. This central role of BSS as a major important confounding factor affecting microbial physiology, significant microbial community rearrangements and interactions with host showcases the importance of the local conditions for transformation of microbial physiology to a causative phenotype [3,12,13,14,15]. Strong connection of stool consistency, i.e., BSS, with microbial gene richness and intestinal inflammation were reported recently for 61 severely obese subjects [16]. Other recent communications [8,9,17,18] focused on BSS stool consistency in (healthy) male and female subjects due to its established correlation with colonic transit time, inflammation, microbiome and metabolome characteristics that are of clinical relevance in numerous gastrointestinal conditions. We recently reported on strong association of BSS, retention time and defecation frequency with increased systemic inflammation, insulin resistance, cardiovascular deconditioning, depression, increased levels of the genus *Bacteroides* and their virulence genes in healthy males after prolonged physical inactivity and hypoxia (PlanHab project) [3,13,14].

The BSS was also shown to demonstrate substantial validity and reliability in general, although difficulties arose around clinical decision points (BSS Types 2/3, 4/5) [19]. Although BSS can be easily evaluated by participants themselves a substantial intra- and inter-rater variance were observed [20,21] due to sensation of straining during defecation. Consequently, self-rating was susceptible to subjective bias despite its effectiveness in clinical use. BSS offered a reliable surrogate measure of stool consistency only when rated by well-trained expert [22]. The non-uniform or mixed makeup of fecal samples, exhibiting two or three or more different BSS classes was identified as another large source of variability in BSS values, preventing the exact classification of mixed form samples and allowing classification errors when ascribing one BSS class to such mixed form samples. These observations point to the need for improved validity and reliability through modifications to the BSS [19].

While retention time, defecation rate, BSS but not water activity have been shown to account for BSS-associated inflammatory effects [3,8,9,13,14,16,17,18] the potential correlation with the strength of a gel [23] in the context of intestinal abrasion forces remains unexplored. In response to external force (stress) complex materials either maintain rigidity, deform semipermanently (viscoelastic materials) or permanently (plastic materials). In the semi-solid materials (i.e., pastes) such as fecal matter minimal pressure (MP; force per unit area) required to induce permanent deformation is proportional to the density of crosslinks with stiffer gels having a higher density of crosslinks [23]. Its importance as a shaping factor for intestinal abrasions and inflammation is currently unclear and has yet to be determined.

In this study, BSS and classical parameters (total solids, water content) [24,25] were recorded for a heterogenous collection of samples and mapped to MP measured in longitudinal and lateral transects of fecal samples. Random samples from prospective male-female study were collected and upgraded with healthy young male samples collected within the controlled four-week bed-rest space-exploration project PlanHab [26]. Gut environment was explored from the perspective of ecosystem development [15] in order to elucidate the relationship between the progressive increase in stool consistency (MP, dry matter) and the progressive intestinal and systemic inflammation observed over the course of the PlanHab project. The MP values reported in this study were coupled to reanalysis of the PlanHab data and fecal 1H-NMR metabolomes reported before [3,13,14] to explore the association between stool consistency (MP), individual signatures in 1H-NMR metabolites and the elevated intestinal, systemic inflammation and other detrimental physiological deconditioning effects observed in the PlanHab participants reported before [3,13,14,26,27,28,29,30,31,32,33,34,35,36,37,38,39,40,41,42,43].

## 2. Results and Discussion

### 2.1. Exploration of the Tripartite Relationship between BSS, Dry Matter Content and Novel MP Values

**Figure 1:**
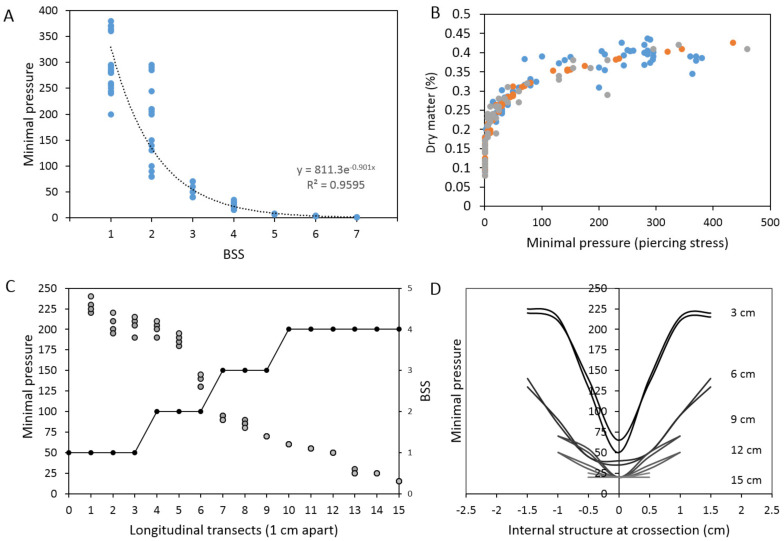
(**A**) Large heterogeneity in stool surface minimal pressure (MP (g/3.14 mm^2^)) was identified within the same BSS class. Note the nonlinear increase in MP. (**B**) Healthy BSS values (3–4) were concentrated around the trade-off between the lowest MP and highest dry matter content. The intermediate density of crosslinks is beneficial for maintenance of human health as based on (•) 78 sample collection and our PlanHab project data (n = 96; [3,13,14]) (•) and (•)). (**C**) An example of stool containing mixed BSS classes (•) and more than an order of magnitude difference in surface minimal pressure (•) along the longitudinal transect of fecal specimen scanned from all 4 sides (n = 4). (**D**) The crossectional (lateral) transects of the same stool example as shown in (**C**) and their respective internal MP values at locations 3, 6, 9, 12 and 15 cm from the fecal tip. A decrease in surface MP and relatively small change in internal MP values can be seen along fecal specimen. Please consult Figures S4 and S5 for more details.

Fecal samples were collected from the prospective male-female random study that served to provide the backbone observations on the tripartite relationship between BSS, dry matter content and novel MP values. This relationship was further amended by superposition of samples collected within the PlanHab project ([3,13,14,26,27,28,29,30,31,32,33,34,35,36,37,38,39,40,41,42,43]; Table S1; Figures S1 and S2) that was designed to capture systemic body deconditioning parameters in response to three-week controlled bed-rest inactivity and hypoxia, a simulation of the space exploration environment. In summary, the decision of the host to reduce physical activity to three-week 24/7 bed-rest resulted in significant increase in insulin resistance, muscle resorption, bone demineralization and other numerous adaptations, hence the complexity of human body physiological responses were collated from the PlanHab literature and summarized in Table S1. The following observations became apparent from the exploration of the tripartite relationship between BSS, dry matter content and MP values:

First, exponential increase in MP [23] values was observed with decreasing BSS (Figure 1A) in 78 fresh stool samples exhibiting a wide array of fecal consistencies (43 males; 35 females) (Spearman r = −0.86, *p* < 0.0001). No significant difference could be detected between male and female samples in this study (*p* > 0.05). Samples within the same BSS class contained highly heterogeneous MP values, giving rise to five times larger variability in observed MP values within the three lowest BSS categories (1–3) (Figure 1A). The same observed relationships between MP and dry matter content were observed also for the PlanHab project samples (n = 92) [3,13,14]. The exponential function (Figure 1A) has little meaning in describing the relationship between MP and BSS beyond establishing the existence of nonlinear relationship in stool hardness (MP) and BSS. These results show that fecal samples exhibit continuous (MP) rather than discrete (BSS) characteristics, hence overcoming the discrete BSS boundaries.

Second, the overall relationship between MP and dry matter content (Figure 1B) for all samples corresponded to rather uniform asymptotic function (y = 0.1343x^0.196^, R^2^ = 0.89). A surprisingly nonlinear, continuous and complex relationship between the two, and the rather disturbing increase in MP at almost negligible increase in observed dry matter content was identified (Figure 1B). Such dependence in fact confirms that the increasing molecular weight of the polymer chain connecting the crosslinks [23] generated the higher density of crosslinks. These results also suggests that at high MP the more rigid gel network, residing for a prolonged time in intestinal environment during constipation, could hardly be remodeled by intestinal muscles (peristaltic waves and segmenting contractions) without any abrasion being inflicted to the soft intestinal mucus and tissues. The correlation between DM and BSS (Figure S3) further confirmed the linear relationship between the two (R^2^ = 0.92) in this study, including the large overlaps between DM in various BSS classes also observed before [3,24]. In this sense (Figure 1A,B) MP was more informative for fine scale and conclusive stratification of stool samples than DM.

Third, the intersection of lowest MP values and highest dry matter content (i.e., the apparent breaking point) corresponded to BSS values designated as healthy (BSS 3–4) (Figure 1B), despite the lower correlation between BSS and dry matter content (Spearman r = −0.76, *p* < 0.0001) in comparison to correlation between MP and dry matter above. This clearly implicates intermediate MP values as highly important for maintenance of microbial activities, mixing and hence maintenance of human health. Beneficial characteristics at such density of gel crosslinks exist at intermediate MP to support non-inflammatory interactions between the microbiome and the host. The further increase in MP at roughly the same dry matter content was highly associated with progressive increase in gut and systemic inflammation reported before [3,13,14] for the same samples in the PlanHab project, showing detrimental effects of excessive crosslinking (Figure 1B).

Four, in this study, the longitudinal mapping of surface MP was performed over the entire length of stool sample to illustrate the difficulty of assigning uniform BSS to complex fecal samples (Figure 1C; Figure S4). These results show that more than an order of magnitude difference in MP can be detected along the length of a single fecal sample (Figure S1) and testifies that MP approach described in this study was able to discern fine grained internal, local differences (four transects over the same sample) that could not have been observed through the sole use of BSS for the whole fecal sample.

Five, lateral transects (Figure 1D) were inspected at 3 cm equidistance over the stool longitudinal transect and showed the existence of resistant surface layer followed by much softer internal structures exhibiting an order of magnitude lower MP values, that were characteristic of healthy, inflammation-free stool consistency (Figure S5).

From measurements obtained in this study it is conceivable that fecal matter is apparently easily mixed by intestinal tract contractions without accompanying negative symptoms characteristic of physical abrasions up to MP < 75. In this study, MP < 75 roughly corresponded to the clinically relevant boundary between BSS 2 and 3, i.e., the boundary between the BSS constipated and BSS normal values, that was so far hard to discern from visual inspection of fecal surface [19]. In contrast, MP > 75 already corresponded to prolonged intestinal transit and signs of constipation [3,13,14] at rather comparable dry matter content. In addition, internal differences in structure could be observed, such as progressively harder surface (e.g., MP~ 300) with the core MP values only twofold higher than in healthy makeup (Figure 1D). On the other hand, MP < 30 corresponded to the category of loose/watery fecal samples in analogy with the degree of stool characteristics described recently (hard/lumpy; normal; loose/watery) [22]. These two MP boundaries (MP < 30; MP > 75; Figure 1B) in fact correspond well to the clinically relevant boundaries for BSS > 4 and BSS < 3, respectively, that have been hard to determine unequivocally by visual inspection of specimen and BSS assignment only. In addition, fecal samples showing characteristics of BSS3 and 4 were shown to likely comprise multiple stool forms mixed together (Figure 1C), increasing thus BSS assignment errors when categorizing such mixed-forms [22]. In this sense, MP approach introduced in this study resolved this problem (Figure 1B) by introducing the continuous scale.

Taken together, in this study we established an exponential relationship between BSS and MP on one side and a complex saturation curve –like relationship between dry matter and MP. The PlanHab samples with decreased BSS values (Figure S1) were reported to be associated with a number of detrimental physiological and psychological characteristics next to intestinal inflammation (Figure S2; Table S1; [3,13,14,26,27,36]). Consequently, the increased stool resistance to remodeling as measured in this study by MP in the PlanHab samples was apparently related to intestinal abrasions, diet associated mucus imprinting and surface pore clogging on stool. These parameters were all shown to exert selective pressure on gut microbiome, its gene expression and metabolic activities, generating thus metabolic makeup associated with observed local and systemic inflammation [3,8,13,14,16], mirrored also in the urinary metabolomes of the same PlanHab project [26]. This clearly showed that parameters other than BSS accurately described clinically relevant fecal hardness.

The relationship observed in this study showed the generalizability and the potential of the MP approach to unequivocally characterize sampling sites in mixed samples (mixed BSS) for future metagenomic and metabolomic studies including mapping of the biochemical nature of the intestinal environment. Within-sample variation is still unresolved problem due to the observed variation between sampling sites in samples with inconsistent structure [44,45]. MP approach resolved this problem with exact characterization of the sampling microlocation. Its small surface area (d = 2 mm; *p* = 3.14 mm^2^) enabled measurements to be uniformly repeated on many different locations over the same fecal sample to provide a multitude of measurements and hence a microscale estimate for particular location or consistency transect of fecal matter, or to map the longitudinal or lateral BSS and MP characteristics of a fecal specimen, hence linking the compactness of material to exact sample location for stratified analyses of metabolomes, host physiology, immune responses or microbiome.

Our results were obtained from samples collected in two independent projects, including males and females, covering a wide span of moisture contents and stool consistencies determined as described before [3,8,9,13,14,16,17,18]. Our data do provide independent evidence for the exponential association between stool consistency and MP as measure of crosslinking (Figure 1) corresponding to the intestinal and also systemic inflammation observed before [3,13,14] (Figures S1 and S2). In addition, simple two dimensional classification by dry matter and MP enables fast mapping of fecal samples for comparisons with unprecedented resolution, surpassing that of user dependent BSS values that have been also criticized for lack of consistency between studies [20].

In this study we presented an approach of minimal pressure (MP) required to induce permanent deformation (piercing) that is proportional to the density of crosslinks with stiffer gels having a higher density of crosslinks. The value of this novel approach comes from the relationship between the stress (compressive loading; forcer per unit area) and strain (deformation) that a particular material exhibits in general. Entangled polymers such as fecal matter are variably characterized by a mixture of physical entanglements between polymer chains and also chemical crosslinks that give the material gel-like properties and can span brittle-ductile material behavior [23]. Different approaches to measurement of material characteristics exist such as penetrometer [46], viscosimeter [47,48] and texture analyzer [25,49,50] and are utilized depending on the necessary sample pretreatment (flattening, homogenization, mixing, averaging, subsampling), complexity of the apparatus (e.g., TA.XTExpressC), measurement approaches (viscosity, stickiness, hardness), and the necessity to record the fine-scale 3D structure of the non-homogenous material specimen. In this sense the MP concept presented in this study and terminology of minimal pressure (MP) represent a significant extension to the existing approaches analyzing stool consistency as site-specific measurements of stool characteristics important for identifying unique metabolome and microbiome signatures are enabled, linking them to exact sampling locations. Further, MP approach does not require any pretreatment (e.g., homogenization, packing, flattening) but enables 3D mapping of the sampling locations relevant for biogeography of intestinal environment. MP approach described in this study provides clinical benefits from being able to more precisely classify BSS group 1, 2 and 3, to better delineate clinically relevant boundaries of consistencies (2/3 BSS; 3/4 BSS), i.e., delineating the central optimal span in MP relevant for the medical delineation between classes.

In addition, the MP approach operates on unmodified fecal sample that can be stored at 4 °C and rewarmed, enables fine-scale longitudinal, crossectional site-specific measurements before actual subsampling for various chemical and molecular analyses, giving rise to descriptions of fecal sample locations relevant for 3D biogeography. Finally, the MP approach does not require sample pretreatment but supports simple direct measurement devoid of complex or expensive apparatus, and hence represents a cheap and operator independent, reproducible and objective alternative that can be utilized globally.

### 2.2. The PlanHab Project Metabolite Signatures Characteristic of High MP

**Figure 2:**
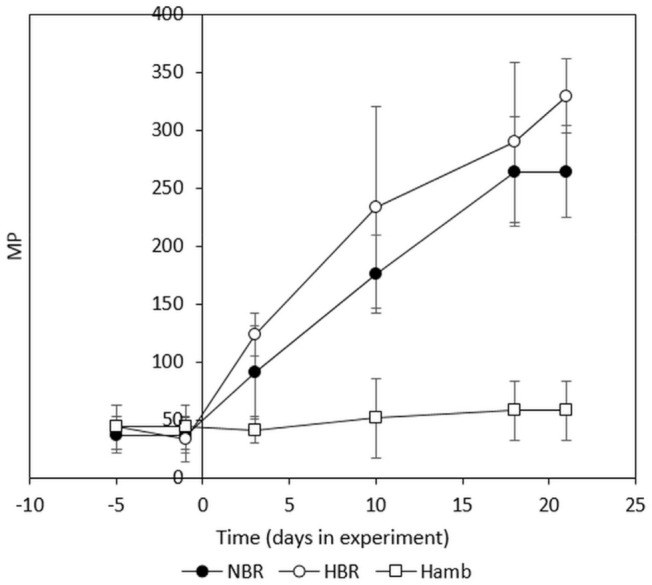
Presentation of MP values for the PlanHab samples in relation to the experimental variants described with BSS (Figure S6). Error bars designate standard deviation. NBR-normoxic bedrest, HBR-hypoxic bedrest, HAmb-hypoxic ambulatory variants of the PlanHab experiment.

MP values recorded for the PlanHab samples were used to show that large variability in stool consistency was hidden within BSS values (Figure 2) recorded for participants [3,13,14,26]. First, these data show the high inter-individual heterogeneity despite the fact that the PlanHab project experiment was conducted under strictly controlled conditions, including diet, immobilization, oxygen level, hydration, circadian rhythm, 24/7 medical surveillance. The three-week experiment under different conditions resulted in progressive body deconditioning (Table S1) next to constipation and increased intestinal and systemic inflammatory responses (Figures S1 and S2). The MP data for the same samples illustrate the profound increase in MP values of progressively constipated participants (Figure 2) that matched with the deconditioning and inflammation markers recorded before (Figure S2). The dose dependent increase in MP in the most affected PlanHab experimental variants illustrated the importance of the MP for the detection of modified intestinal conditions, characterized by five to six times higher MP, previously linked to the access of pathogens and endotoxins to the epithelium through physical mucus compaction [51] and abrasions due to long-term residence and regular intestinal muscle contractions [3,13,14].

Second, to further explore the utility of MP strategy in metabolomic analyses of intestinal tracts, MP was utilized in reanalysis of our previously published fecal 1H-NMR metabolomes from the same samples obtained from the 4-week PlanHab project [14] (Table S1). The corresponding 1H-NMR data were reanalyzed using the latest ChenomX 8.6 software and linked to MP data collected for the same samples. Principal coordinate analysis of the PlanHab 1H-NMR metabolomes showed the existence of rather unique and highly individualized metabolic signatures over the course of the PlanHab experiment (Figure S6). Essentially, each sample received unique MP value, showcasing the much finer and continuous resolution for the locations from which the samples were collected. Consequently, much larger numbers of samples would need to be collected to model MP relative to complexity of 1H-NMR metabolomes. Power analysis estimates suggested two orders of magnitude larger sample size in the range of 10.000 would be required as observed in recent metagenomic studies [52,53].

**Figure 3:**
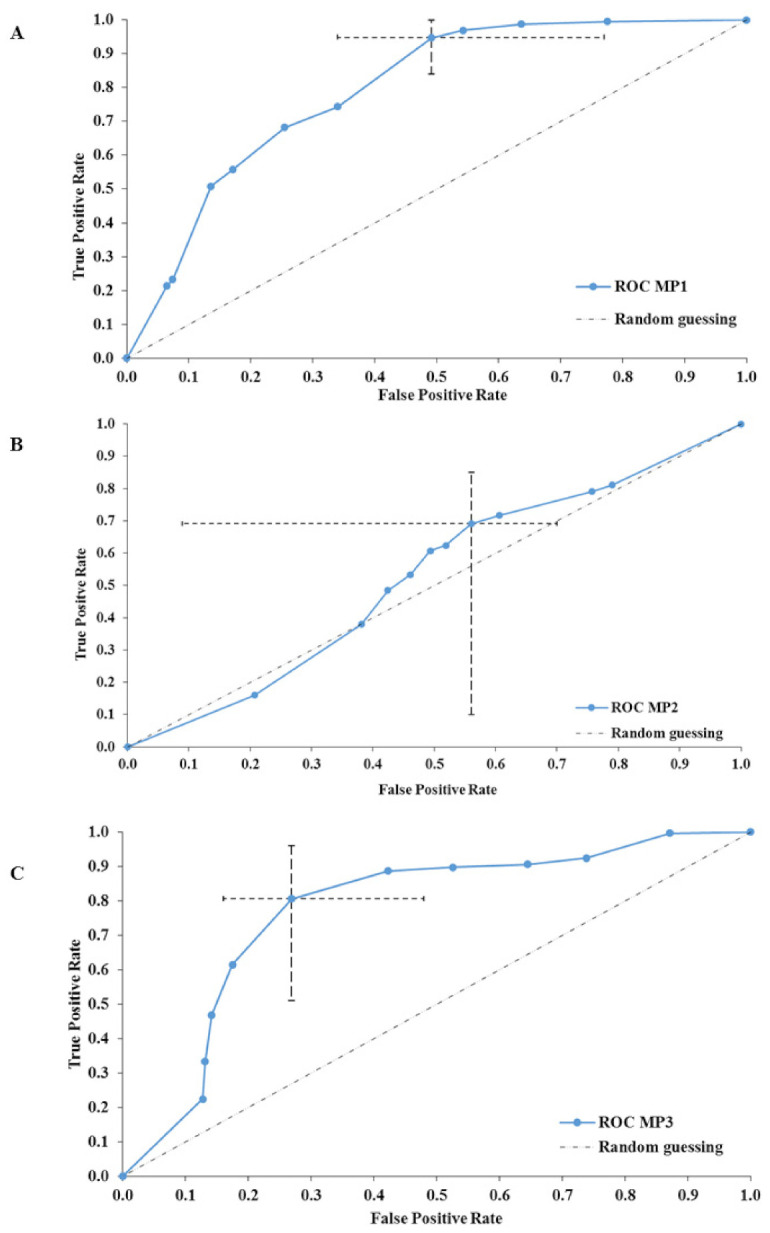
An overview of the model performance in three different groups between fecal metabolomes in delineation with three different groups of MP (MP1 < 30 (**A**), 30 < MP2 < 75 (**B**) and MP3 > 75 (**C**)). Please note the rather uncharacteristic and hard to classify makeup of metabolomes observed in the intermediate, healthy, group (**B**). Horizontal and vertical dashed lines represent 95% confidence interval for false positive and true positive rates, respectively. The black dashed line represents model performance in case of random guessing; blue line represents training mean performance of the model.

Third, when the same 1H-NMR data were utilized on more coarse scale to explore the more recent classification [22] with the loose/watery, normal/healthy and hard/lumpy classification [22], the two MP boundaries identified in this study (MP < 30; MP > 75; Figure 1B) matched the clinically relevant boundaries for BSS < 3 and BSS > 4, giving rise to three rather broad categories of MP classes: MP1 < 30; 30 < MP2 < 75; MP3 > 75. These classes further match with the loose/watery normal/healthy and hard/lumpy classification [22] and were used in machine learning and modeling in search of significant differences in metabolomics. Analyses utilizing BSS assignments were described before [14]. Ridge logistic regression model with penalty hyperparameter 1.0 as the predictive algorithm was selected as the best interpretable model (AUC = 0.783). Constant Removal and standardization and LASSO feature selection (Penalty = 1.0, Lambda = 1.558e-01) were used in this context. The output of selected features with LASSO regression was used for prediction with ridge logistic regression model. Both were chosen with automatic machine learning process as the best option form 168952 trained models. Acetate and methionine were selected as reference signatures from the 174 analyzed metabolites. According to Individual Conditional Expectation (ICE) plots, increased concentrations of acetate increased the probability of such a sample being classified in the low minimum pressure group, while increased concentrations of methionine increased the likelihood of such a sample being classified as the medium to high MP group. The 95% CI of the model performance achievable by using acetate alone ranged from 94.1 to 100%. The addition of methionine to the analysis, the model performance was effectively close to 100%. The best interpretable model was validated on the test data, selecting acetate and methionine as predictive features, and with AUC = 0.833, the validation of the model was successful. According to the receiver operating characteristic curves (ROC), the model showed better performance in classifying in MP1 and MP3 groups (Figure 3A,C), with lower or higher MP values. On the other hand, the performance was lowest in MP2 group (Figure 3B). This further showcases the large inter-individual differences and variable responses to the same diet in the PlanHab participants. Power analysis showed that two orders of magnitude larger samples would be needed to effectively build submodels for the metabolic diversity of the apparently healthy gut metabolomes.

Taken together, metabolomes belonging to MP1 and MP3 groups outside the central span of MP2 values possessed sufficient information for acceptable sample classification. Surprisingly, the makeup of the intermediate MP2 group did not exhibit any characteristic signatures (Figure 2B). A wide array of metabolic makeups mirroring characteristic of inter-individual differences in physiological makeup of healthy microbiomes in feces were reported before [12]. From this it follows that lower and upper extremes (i.e., MP1 or MP3) contained features distributed characteristically enabling their separation. JADBIO machine learning and modelling [54] showed that out of all measured metabolites, the two most important features responsible for separation of the three groups were acetate, a short chain fatty acid, produced by microbes, and methionine, a compound involved in regulation of metabolic processes, the innate immune system, digestive functioning in mammals next to their lipid metabolism, activation of endogenous antioxidant enzymes (methionine sulfoxide reductase A), and the biosynthesis of glutathione to counteract oxidative stress [55]. Finally, methionine restriction was shown to decrease DNA damage and carcinogenic processes, averting arterial, neuropsychiatric, and neurodegenerative disease [55]. Fecal acetate was shown to be inversely related to acetate absorption from the human colon, and high circulating acetate concentrations were negatively correlated to insulin sensitivity [56]. In our past analyses of the PlanHab urine 1H-NMR metabolomes [26] elevated acetate concentrations were observed in most constipated participants, exhibiting insulin resistance, modified fat oxidation, bone demineralization, muscle deconditioning and depression (Table S1; Figure S1; Figure S2) [27,34,39,40]. The fact that the PlanHab data was derived from a medically prescreened cohort receiving defined and synchronized diet, characteristic of Western Diet [3,27,34] in tightly controlled environment of the 4-week PlanHab study (levels of exercise, circadian rhythm, medical care, oxygen pressure) enabled us to study the existence of significant differences between fecal metabolomes and exact fecal makeup at the sampling location (MP), rather than average BSS assignment (Figure S1) described before [3]. Therefore the observed differences in the acetate and methionine levels in fecal samples stem from the conserved differences in their uptake as a result of responses to inactivity, coupled with Western type of the diet utilized in the PlanHab project.

The inclusion of the newly described parameters such as MP, acetate and methionine extends our previous findings on the significant parameters associated with detrimental effects of inactivity on human body [3,13,14,26] and its mechanisms: the decision of the host to reduce physical activity gave rise to increased fecal electrical conductivity that lead to decreased BSS (constipation) and increased bile acids (BA) levels, while at the same time reduced indole concentrations at retained high electrical conductivity resulted in higher intestinal inflammation (EDN) levels (Figure S2). The relationships observed in Bayesian modelling [14] identified that nonlinear responses take place over the network as small changes in indole levels exhibited unexpectedly large effects on BA content. Taken together, this clearly shows that introduction of nonlinear MP as an extension to BSS and observing an increasing MP in fecal samples describing fecal surface deformability, represents the link between physical abrasions due to intestinal muscle contractions, microbial indole and acetate production and intestinal inflammation marker EDN reported before [3,13,14,26].

## 3. Materials and Methods

### 3.1. Fecal Sample Collection and Analysis

Samples utilized in analyses in this study were collected within the prospective study (n = 78) and the PlanHab project (n = 96), anonymized by coding following collection and subsequently characterized for various physical and chemical characteristics as described before [3,13,14,26]. All participants gave written informed consent after receiving detailed information regarding the study protocol and all experimental procedures. Ethics Committee permission was obtained from the National Ethics Committee of the Republic of Slovenia and is held by Jožef Stefan Institute.

The participants of the prospective study represented a one-time sampling cohort spanning 78 fresh stool samples exhibiting a wide array of fecal consistencies of otherwise healthy nonsmoking 43 males and 35 females. The participants of the prospective study were represented by healthy, non-obese (BMI < 30 kg/m^2^) males and females (22–42 years). Baseline male and female characteristics were as following; age (28 ± 5 years and 29 ±4 years); body mass (76 ± 10.5 kg and 61± 8 kg), BMI (23 ± 5 kg/m^2^ and 23± 3 kg/m^2^), respectively.

Further, the PlanHab healthy male participants were characterized by numerous clinically relevant measurements to assert absence of disease with a state of physical, mental, and social welfare. Their baseline characteristics were as following; age (27 ± 6 years); body mass (76.7 ± 11.8 kg), BMI (23.7 ± 3 kg/m^2^) [27,34]. The participants underwent 5 days of baseline data collection during which participants were ambulant, 21 intervention days and 5–14 days of medical follow-up. The PlanHab project participants (n = 11) were provided with an individually tailored, standardized, and controlled diet throughout the intervention as described before [3,27,34]. Energy requirements were assessed with the Harris-Benedict method, and correction factors of 1.4 and 1.2 were used to account for activity levels in the ambulatory phases and the bed rest phases, respectively. In addition to a controlled intake of fat (30%) and protein (1.2 g per kg body mass), sodium intake was set to 3500 mg per day. Participants were supplemented with 1000 IU vitamin D3 per day. Fluid intake was ad libitum, but participants were encouraged to drink at least 28.5 mL per kg per day. Importantly, menu plans were cycled in the same way for each participant across the three experimental conditions, adjusting the quantity according to activity factors above. The collected fecal samples (Figure S1) thus represent a longitudinal transect where intestinal tracts developed progressive constipation and a number of systemic physiological deconditioning symptoms (Figure S2) [3,13,14,26]. In total, 96 samples were collected over the course of the PlanHab experiment for 11 participants. Their data relevant for this study (BSS, inflammation (eosinophile derived neurotoxin, systemic inflammation) are presented in Figures S1 and S2.

The BSS score was assigned immediately after the collection of specimen as before [3,10,11,13,14].

Water content of the fresh sample was determined by collecting samples (~70 g) into pre-weighted 200 mL collection jars and sample mass determined by second weighing of the jar. Water content of a sample was determined by drying at 60 °C for 48 h. The sample was cooled in a laboratory desiccator and weighed again. The water content of a sample was calculated as the difference in final and initial mass of the sample, divided by the initial mass [57]. The dry matter content of the fresh sample was calculated by subtracting water content from 1.

### 3.2. Measurements of Minimal Pressure

Here, we measure minimal pressure of 170 fecal samples with different BSS consistencies using a defined flat-cut stainless steel probe (d = 2 mm; s = 3.14 mm^2^) with attachable adjustable weights (1–400 g) at constant temperature (25 °C). Probe indentation [23] by gravity that is independent of probe velocity tests primarily the response of the gel network and hence the measured hardness shows a strong dependence on the increasing molecular weight of the polymer chain connecting the crosslinks [23] generating the higher density of crosslinks [23].

In practice the approach is used to determine the minimum weight per unit area needed to pierce through the surface of fecal specimen. The utility was developed in a way that additional weights were attached to the stainless steel rod until the weight was sufficient to pierce through the material. Due to its small surface area the MP measurements were uniformly repeated on many different locations over each specimen. This enabled us to provide a multitude of measurements and hence a microscale estimate for particular location of fecal specimen. Longitudinal transects were probed 0.5 cm apart, then the specimen was rotated for 90° and measured again. Lateral transects were obtained after the specimen was cut at locations of 3 cm, 6 cm, 9 cm, 12 cm and 15 cm. Readings were recorded for two perpendicular transects within each specimen.

The MP measurements at 37 °C were in general 10% lower in comparison to those determined at 24 °C. In addition, MP measurements taken within the 24 and 48 h stored at 4 °C and measured at 24 °C after 60 min reheating were not significantly different.

Principal coordinate analysis was conducted on Box-Cox transformed metabolomics data and Benjamini-Hochberg significance correction for multiple comparisons was used in non-parametric npMANOVA as described before [3,13].

### 3.3. Intestinal Metabolome Analysis Using Proton Nuclear Magnetic Resonance (1H-NMR)

The 1H-NMR intestinal metabolomic data published before [14] were reanalyzed using a novel version of ChenomX 8.6 and analyzed utilizing machine learning approach (Just Add Data Bio (JADBIO, version 1.1.182)) [54].

In essence, the reanalyzed data were obtained as described before [14]: fecal samples (200 mg of dry matter) were resuspended in 800 μL of NMR phosphate buffer and centrifuged at 10,000 *g* for 30 min at 4 °C to remove fine particles. Samples were filtered through 0.22 μm HPLC compatible filters (Millipore, Germany), 400 μL aliquots were mixed with 200 uL 1H-NMR buffer as described before [58] and stored at −25 °C until analysis. Phosphate buffer (pH 7.4) was prepared by weighing 1.443 g Na_2_HPO_4_, 0.263 g NaH_2_PO_4_, 2 mM TSP, and 1 mM NaN_3_ into 50 mL volumetric flask. Ten milliliter of D_2_O was added and filled up to 50 mL with Milli-Q water. Before analysis, samples were thawed at room temperature, centrifuged at 12,000 g for 5 min at 4 °C. In total, 550 μL of each sample was transferred into 5 mm NMR tube.

1H-NMR spectra were acquired on an Agilent Technologies DD2 600 MHz NMR spectrometer equipped with 5 mm HCN Cold probe. The 2D experiments were measured on Agilent Technologies (Varian) VNMRS 800 MHz NMR spectrometer equipped with 5 mm HCN Cold probe. All experiments were measured at 25 °C. 1H-NMR spectra of the samples were recorded with spectral width of 9.0 kHz, relaxation delay 2.0 s, 32 scans and 32 K data points. Water signal was suppressed using Double-pulsed field gradient spin echo (DPFGSE) pulse sequence. Heteronuclear single quantum coherence spectrum (HSQC) was acquired for 1H and 13C dimensions and total correlated spectrum (TOCSY) was measured with 1H spectral widths of 7.0 kHz, relaxation delay 1.5 s, 160 number of transient and 128 time increments. For apodization of acquired spectra, we used exponential and a cosine-squared functions. NMR spectra were processed using VMRJ (Agilent/Varian) and Sparky (UCSF) software and MestReNova.

The resulting spectra were consequently analyzed using targeted quantitative metabolomics using Chenomx NMR Suite version 8.6 (2020; Chenomx, Canada). All spectra were randomly ordered for spectral fitting using ChenomX profiler. Metabolites analyzed in this study were identified using the support of Chenomx Compound Library extended by Human Metabolome Data Base [59].

Automated Machine Learning was used to identify most important metabolic features separating the groups of fecal matter plasticity. Samples were divided into three separate groups indicating low ((MP1 < 30), medium (30 < MP2 < 75), and high (MP3 > 75) MP, and 174 metabolic features were analyzed for possible differentiation. JADBIO version 1.1.182 was used for model generation. Data were split in a 70:30 ratio for model training (70%) and model validation (30%). An extensive tuning effort with six CPU cores was used to compute the most interpretable classification model, which was selected based on the area under the curve (AUC) metric among 168952 trained models. Different algorithms with different combinations of tuned parameters were used for feature selection (LASSO regression and test-budgeted statistically equivalent signature) and for prediction (ridge logistic regression, support vector machines, classification random forest and classification trees). Metabolic data were preprocessed with constant removal and standardized. LASSO feature selection (penalty = 1.0, lambda = 1.558e-01) was used for metabolite feature selection. The output of feature selection was used for obtaining the best interpretable model using the predictive ridge logistic regression algorithm (with hyper-parameter penalty equal to 1.0).

The machine learning process described in this study was adopted for several reasons: (i) automation in parameter and algorithm selection results in reduced bias and human interference; (ii) the approach includes several different ML algorithms (linear regression, SVM, decision tree, random forest and Gaussian kernel SVMs and automatically choose the most interpretable model based on AUC metric; (iii) the resulting models were trained with different configurations on different sub-samples of the original dataset (cross- validation); (iv) focus on relevant humanly interpretable models. Consequently, algorithm, hyperparameter and space selection (AHPS) as implemented in JADBIO was used for selecting the most suitable algorithm for preprocessing and transformation of a given dataset, its feature selection and modeling. The output of AHPS step was analyzed by configuration evaluation protocol (CEP) in order to find the optimal configuration reported in this study [54,60].

To evaluate model classification, a receiver-operating characteristic curve (ROC curve) was constructed for all three groups, plotting the true-positive rate (sensitivity) against the false-positive rate (1-specificity). Individual conditional expectation (ICE) plots revealed the nature of the contribution of each metabolite feature to the model.

## 4. Conclusions

In this study, a minimal pressure (MP) approach utilizing probe indentation of intact fecal samples was introduced as a measure of stool consistency. MP values recorded over a spectrum of moisture contents increased exponentially relative to BSS and enabled stratification of samples exhibiting mixed BSS classes. A trade-off between lowest MP and highest dry matter content delineated the span of intermediate healthy density of gel crosslinks. The crossectional transects identified fecal surface layers with exceptionally high MP suggestive of mucus imprinting overlying internal fecal structures with an order of magnitude lower MP characteristic of healthy stool consistency. The exponential association between stool consistency and MP determined in this study was mirrored in the elevated intestinal and systemic inflammation next to other detrimental physiological deconditioning effects observed in the PlanHab participants reported before. High inter-individual differences in fecal 1H-NMR metabolomes derived from a wide spectrum of MP showed the importance of the exact sampling location in future microbiome and metabolome studies. In conclusion, we believe that the MP approach described in this study it can be used to better understand fecal hardness and its relationships to human health as it provides a simple, fine scale and objective stool classification approach.

## Data Availability

BSS, minimal pressure, dry matter content data next to 1H-NMR fecal metabolomes of the PlanHab project are made available upon request to the corresponding author. Model with test data for clustering samples according to metabolite profiles are included in the supplementary material. Instructions for running a model on local machine are included in the electronic supplementary material.

## Supplementary Materials

The following are available online at https://www.mdpi.com/2218-1989/11/3/172/s1, Figure S1: Changes in Bristol stool scale values (A) and retention time (as time between particular defecations) (B) during run-in (week 1) and subsequent 3-week experimental phase of the PlanHab project, Figure S2: Heatmap plot showing the relationship between parameters describing human physiology, psychology, and intestinal environment that differed significantly at the end of the PlanHab experiment, Figure S3: A scatter plot depicting the relationship between dry matter (% DM) and BSS class assignments, Figure S4: Schematic representation of BSS and MP along the longitudinal transect of fecal specimen, Figure S5: A schematic representation of crossectional (internal) MP values for fecal bolus (upper) and along the longitudinal transect of fecal specimen of mixed type, for which a uniform BSS classification is ambiguous, Figure S6: Principal Coordinate Analysis of fecal 1H-NMR metabolomes sampled over the course of the 4-week PlanHab project, Table S1: A compilation of the PlanHab project publications containing relevant sources of information.
