# Supplementary material for: The Importance of Objective Stool Classification in Fecal 1H-NMR Metabolomics: Exponential Increase in Stool Crosslinking Is Mirrored in Systemic Inflammation and Associated to Fecal Acetate and Methionine
Source: Metabolites. 2021 Mar 16;11(3):172. doi: 10.3390/metabo11030172 (PMC8002301; doi:10.3390/metabo11030172)
Supplement: Supplementary file 1 [file metabolites-11-00172-s001.pdf]

Electronic supplementary material

The importance of objective stool classification in fecal 1H-NMR metabolomics: exponential increase in stool crosslinking is mirrored in systemic inflammation and association to fecal acetate and methionine

Leon Deutsch[1], Blaž Stres[1,2,3,4]

[1] University of Ljubljana, Biotechnical Faculty, Jamnikarjeva 101, SI-1000 Ljubljana, Slovenia

[2] University of Ljubljana, Faculty of Civil and Geodetic Engineering, Jamova 2, SI-1000 Ljubljana, Slovenia

[3] Jožef Stefan Institute, Department of Automation, Biocybernetics and Robotics, Jamova 39, SI-1000 Ljubljana, Slovenia

[4] University of Innsbruck, Department of Microbiology, Technikerstrasse 25d, A-6020 Innsbruck, Austria

Correspondence to Blaz Stres, University of Ljubljana, Biotechnical Faculty, Group for Microbiology and Microbial Biotechnology, Jamnikarjeva 101, 1000 Ljubljana, Slovenia. Email: blaz.stres@bf.uni-lj.si; Phone: +38641567633, Fax: +386 1 724 10 05.

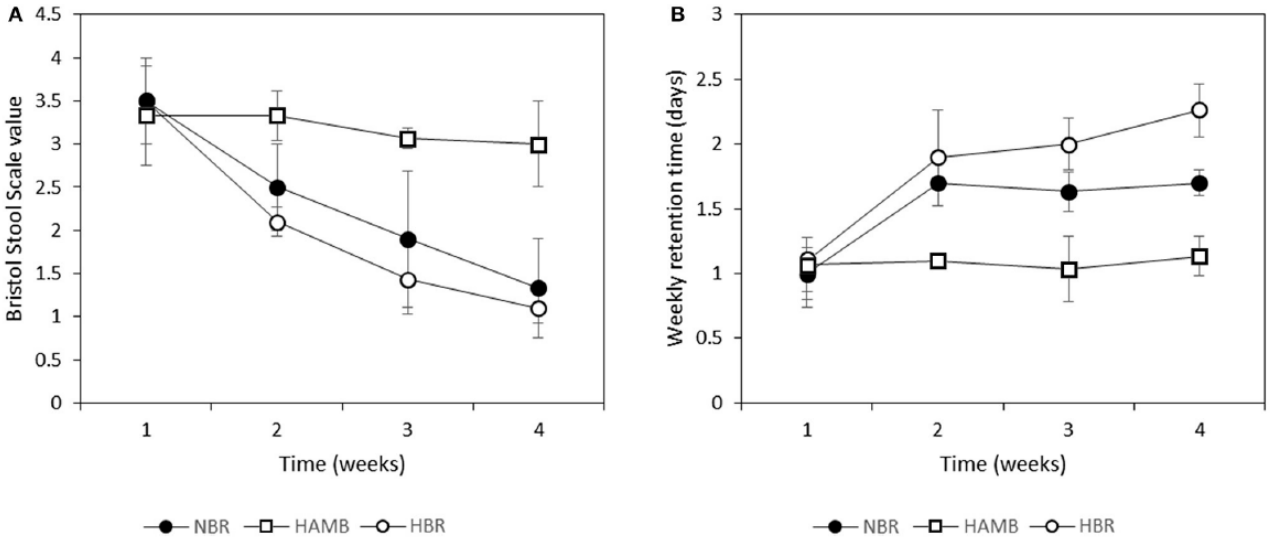

Figure S1: Changes in Bristol stool scale values (A) and retention time (as time between particular defecations) (B) during run-in (week 1) and subsequent 3-week experimental phase of the PlanHab project. Error bars designate standard deviation. NBR- normoxic bedrest; HBR- hypoxic bedrest;. HAmb – hypoxic ambulatory experimental variant.

(Reproduced from Sket et al., 2017a. Authors publishing with Frontiers have their articles under the Creative Commons licence CC-BY which permits re-use, distribution and reproduction of material from published articles, provided the original authors and source are credited [1]).

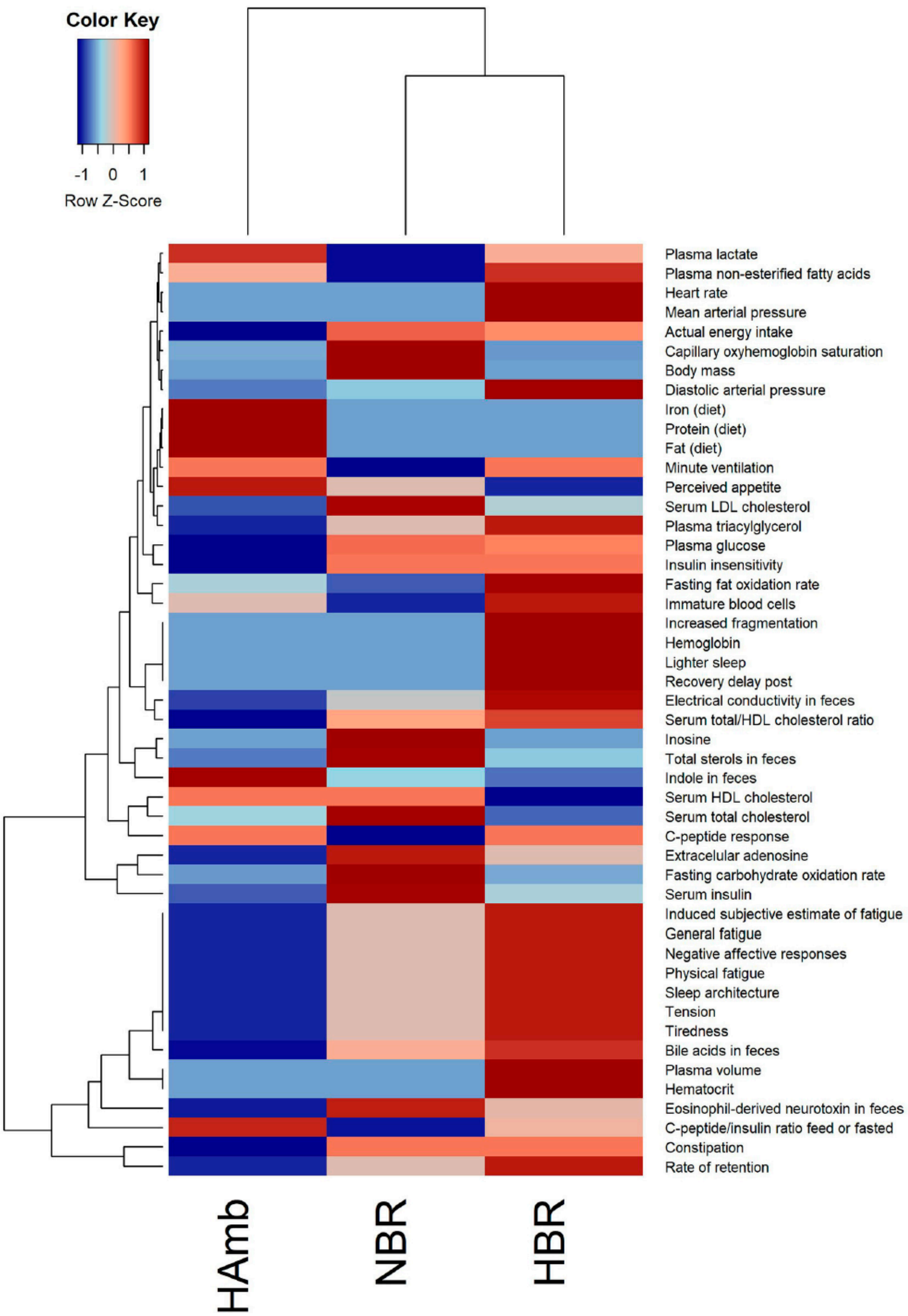

Figure S2: Heatmap plot showing the relationship between parameters describing human physiology, psychology, and intestinal environment that differed significantly at the end of the PlanHab experiment (n = 48; $p < 0.05$; FDR corrected) that are now part of the new version of the in-house PlanHab database (Sket et al., 2017b) based on all measured variables within the project. The inset to the left represents the magnitude of z-normalized data. NBR- normoxic bedrest; HBR- hypoxic bedrest;. HAmb – hypoxic ambulatory experimental variant.

(Reproduced from Sket et al., 2020. Authors publishing with Frontiers have their articles under the Creative Commons licence CC-BY which permits re-use, distribution and reproduction of material from published articles, provided the original authors and source are credited [2]).

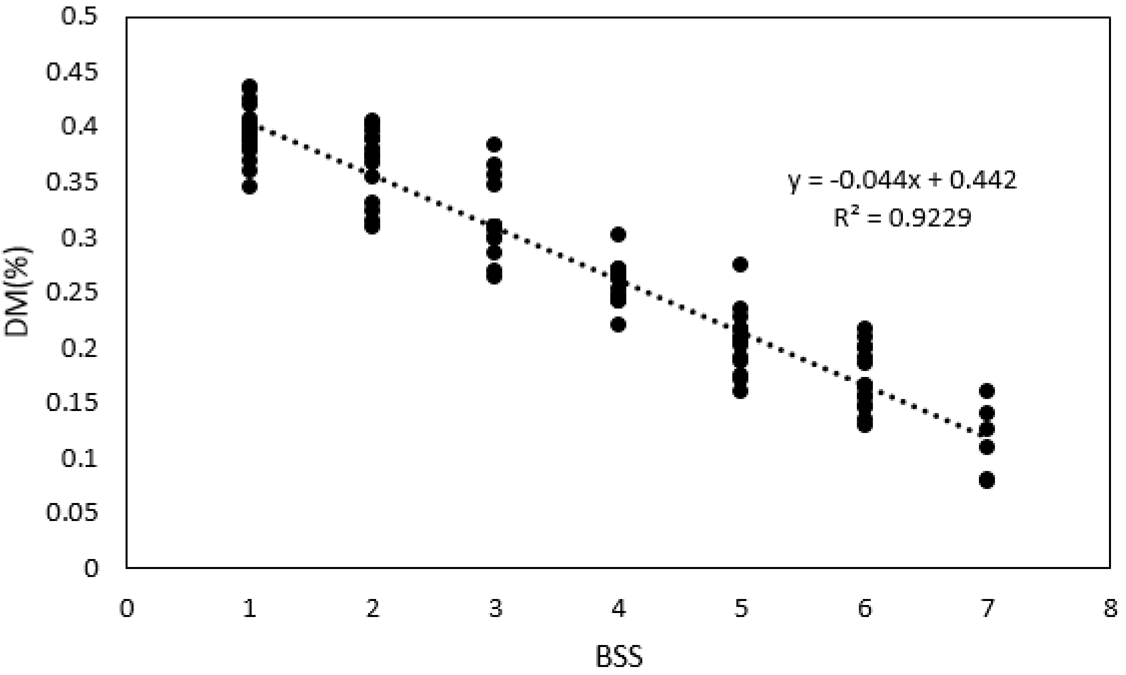

Figure S3: A scatter plot depicting the relationship between dry matter (% DM) and BSS class assignments. Please note the large overlaps in DM content between different BSS classes showcasing that DM content is not definite descriptor of stool consistency and classification. Please consult manuscript Figure 1 for more details.

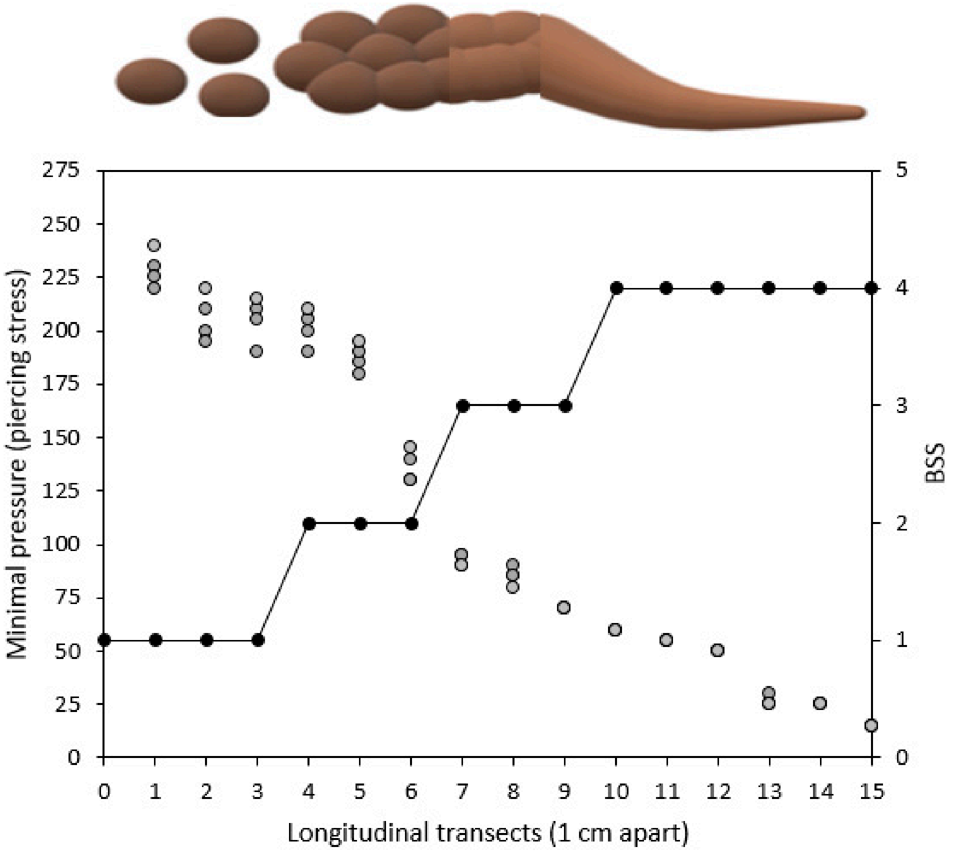

Figure S4: Schematic representation of BSS and MP interplay along the longitudinal transect of fecal specimen of mixed type, for which a uniform BSS classification is ambiguous.

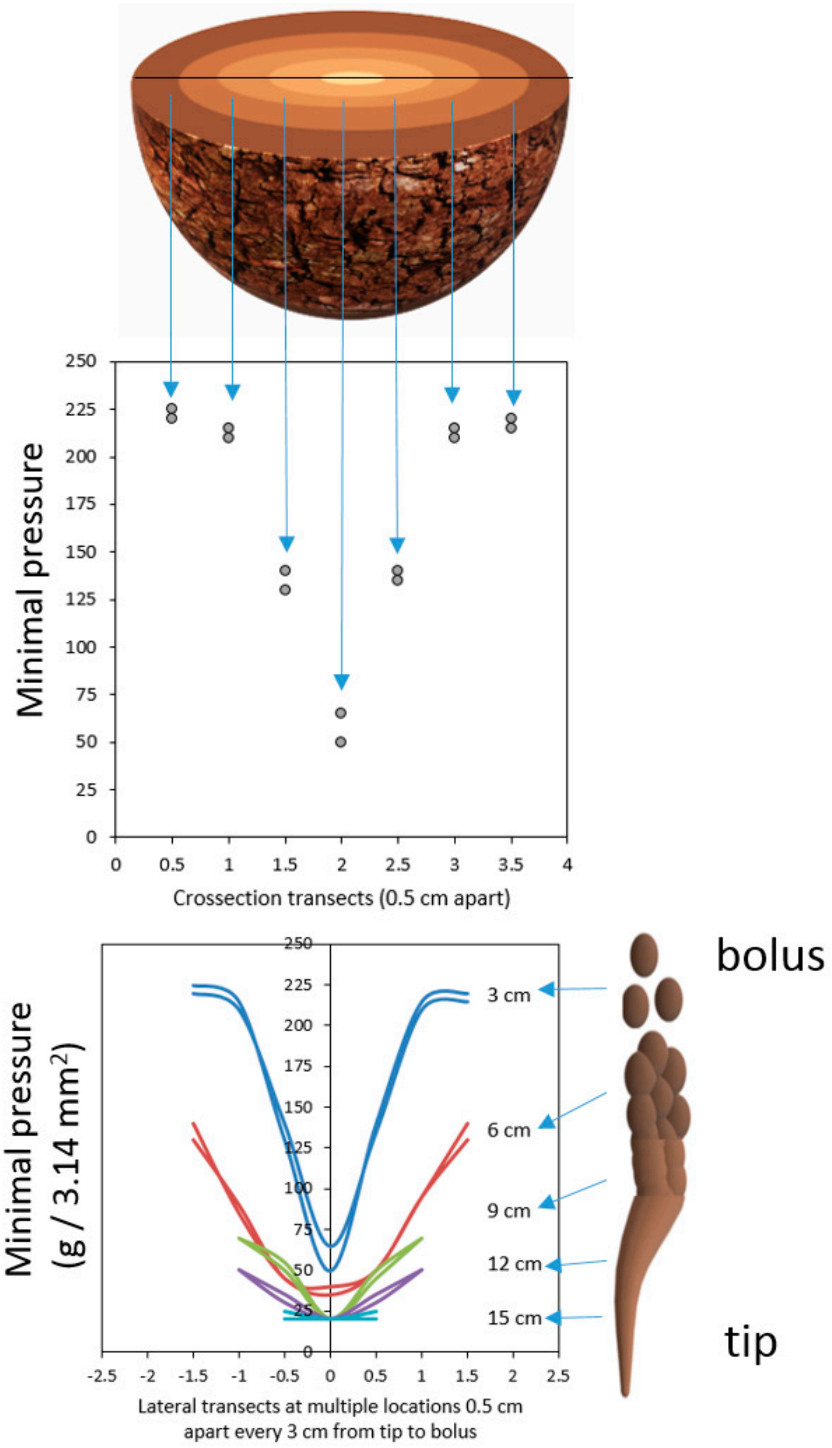

Figure S5: A schematic representation of crossectional (internal) MP values for fecal bolus (upper) and MP values along the longitudinal transect of fecal specimen of mixed type, for which a uniform BSS classification is ambiguous (lower). Please note the existence of softer internal core and progressively harder surface from tip to bolus.

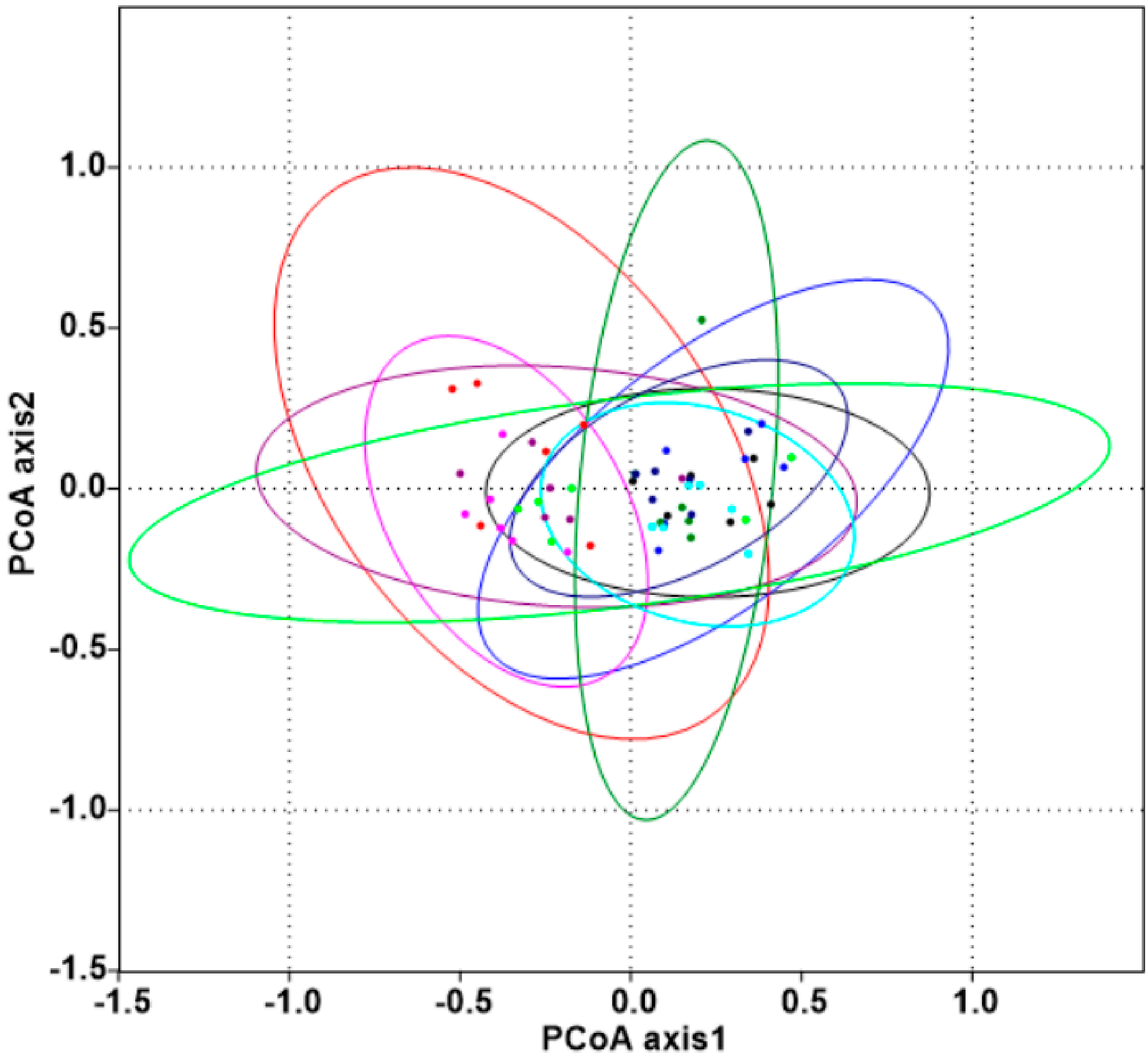

Figure S6: Principal Coordinate Analysis of fecal 1H-NMR metabolomes sampled over the course of the 4-week PlanHab project utilizing Raup-Crick distance with 95% concentration ellipses designating the size of data scatter within each participant.

Table S1. A compilation of the PlanHab project publications containing relevant sources of information.

Ref	Abstract
(Debevec et al., 2014) [3]	**Purpose:** This study tested the hypothesis that hypoxia exacerbates reductions in body mass observed during unloading. **Methods:** To discern the separate and combined effects of simulated microgravity and hypoxia, 11 healthy males underwent three 21-day campaigns in a counterbalanced fashion: (1) normoxic bed rest (NBR; FiO_2 = 0.209; PiO_2 = 133.1 ± 0.3); (2) hypoxic ambulatory confinement (HAMB; FiO_2 = 0.141 ± 0.004; PiO_2 = 90.0 ± 0.4; ~4,000 m); and (3) hypoxic bed rest (HBR; FiO_2 = 0.141 ± 0.004; PiO_2 = 90.0 ± 0.4). The same dietary menu was applied in all campaigns. Targeted energy intakes were estimated individually using the Harris-Benedict equation taking into account whether the subjects were bedridden or ambulatory. Body mass and water balance were assessed throughout the campaigns. Whole body and regional body composition was determined before and after the campaigns using dual-energy X-ray absorptiometry. Before and during the campaigns, indirect calorimetry and visual analogue scores were employed to assess the resting energy expenditure (REE) and perceived appetite sensations, respectively. **Results: Energy intakes were lower than targeted in all campaigns (NBR: -5%; HAMB: -14%; HBR: -6%; P < 0.01). Body mass significantly decreased following all campaigns (NBR: -3%; HAMB: -4%; HBR: -5%; P < 0.01). While fat mass was not significantly altered, the whole body fat free mass was reduced (NBR: -4%; HAMB: -5%; HBR: -5%; P < 0.01), secondary to lower limb fat-free mass reduction. Water balance was comparable between the campaigns.** No changes were observed in REE and perceived appetite.
(Debevec et al., 2016) [4]	Environmental hypoxia and inactivity have both been shown **to modulate appetite**. To elucidate the independent and combined effects of hypoxia and bed rest-induced inactivity on appetite-related hormones and subjective appetite, eleven healthy, non-obese males underwent three experimental interventions in a cross-over and randomized fashion: 1) Hypoxic confinement combined with daily moderate-intensity exercise (HAMB, FiO2 = 0.141 +/- 0.004; PiO2 = 90.0 +/- 0.4 mmHg) 2) Bed rest in normoxia (NBR, FiO2 = 0.209; PiO2 = 133.1 +/- 0.3 mmHg) and 3) Bed rest in hypoxia (HBR, FiO2 = 0.141 +/- 0.004; PiO2 = 90.0 +/- 0.4 mmHg). A mixed-meal tolerance test (MTT), followed by an ad libitum meal were performed before (Pre) and after 16-days (Post) of each intervention. Composite satiety scores (CSS) during the MTT were calculated from visual analogue scores, while fasting and postprandial concentrations of total ghrelin, peptide YY (PYY), glucagon-like peptide-1 (GLP-1) and leptin were quantified from arterialized-venous samples. **Postprandial CSS were significantly lower at Post compared to Pre in NBR only (P < 0.05) with no differences observed in ad libitum meal intakes. Postprandial concentrations and incremental area under the curve (AUC) for total ghrelin and PYY were unchanged following all interventions. Postprandial GLP-1 concentrations were only reduced at Post following HBR (P < 0.05) with resulting AUC changes being significantly lower compared to HAMB (P < 0.01). Fasting leptin was reduced following HAMB (P < 0.05) with no changes observed following NBR and HBR.** These findings suggest that independently, 16-day of simulated altitude exposure (approximately 4000 m) and bed rest-induced inactivity do not significantly alter subjective appetite or ad libitum intakes. The measured appetite-related hormones following both HAMB and HBR point to a situation of hypoxia-induced appetite stimulation, although this did not reflect in higher ad libitum intakes.
(Keramidas et al., 2016) [5]	The study examined the effects of hypoxia and horizontal bed rest, separately and in combination, on peak oxygen uptake (Vo2 peak) during upright cycle ergometry. Ten male lowlanders underwent three 21-day confinement periods in a counterbalanced order: 1) normoxic bed rest [NBR; partial pressure of inspired O2 (PiO2) = 133.1 +/- 0.3 mmHg]; 2) hypoxic bed rest (HBR; PiO2 = 90.0 +/- 0.4 mmHg), and 3) hypoxic ambulation (HAMB; PiO2 = 90.0 +/- 0.4 mmHg). Before and after each confinement, subjects performed two incremental-load trials to exhaustion, while inspiring either room air (AIR), or a hypoxic gas (HYPO; PiO2 = 90.0 +/- 0.4 mmHg). Changes in regional oxygenation of the vastus lateralis muscle and the frontal cerebral cortex were monitored with near-infrared

	spectroscopy. Cardiac output (CO) was recorded using a bioimpedance method. **The AIR Vo2 peak was decreased by both HBR (approximately 13.5%; P </= 0.001) and NBR (approximately 8.6%; P </= 0.001), with greater drop after HBR (P = 0.01). The HYPO Vo2 peak was also reduced by HBR (-9.7%; P </= 0.001) and NBR (-6.1%; P </= 0.001). Peak CO was lower after both bed-rest interventions, and especially after HBR (HBR: approximately 13%, NBR: approximately 7%; P </= 0.05). Exercise-induced alterations in muscle and cerebral oxygenation were blunted in a similar manner after both bed-rest confinements. No changes were observed in HAMB.** Hence, the bed-rest-induced decrease in Vo2 peak was exaggerated by hypoxia, most likely due to a reduction in convective O2 transport, as indicated by the lower peak values of CO.
(Louwies et al., 2016) [6]	Purpose: To assess the separate and combined effects of exposure to prolonged and sustained recumbency (bed rest) and hypoxia on retinal microcirculation. Methods: Eleven healthy male subjects (mean +/- SD age = 27 +/- 6 years; body mass index [BMI] = 23.7 +/- 3.0 kg m-2) participated in a repeated-measures crossover design study comprising three 21-day interventions: normoxic bed rest (NBR; partial pressure of inspired O2, PiO2 = 133.1 +/- 0.3 mm Hg); hypoxic ambulation (HAMB; PiO2 = 90.0 +/- 0.4 mm Hg), and hypoxic bed rest (HBR; PiO2 = 90.0 +/- 0.4 mm Hg). **Central retinal arteriolar (CRAE) and venular (CRVE) equivalents were measured at baseline and at regular intervals during each 21-day intervention.** Results: Normoxic bed rest caused a progressive reduction in CRAE, with the change in CRAE relative to baseline being highest on day 15 (DeltaCRAE = -7.5 mum; 95% confidence interval [CI]: -10.8 to -4.2; P < 0.0001). Hypoxic ambulation resulted in a persistent 21-day increase in CRAE, reaching a maximum on day 4 (DeltaCRAE = 9.4 mum; 95% CI: 6.0-12.7; P < 0.0001). During HBR, the increase in CRAE was highest on day 3 (DeltaCRAE = 4.5 mum; 95% CI: 1.2-7.8; P = 0.007), but CRAE returned to baseline levels thereafter. Central retinal venular equivalent decreased during NBR and increased during HAMB and HBR. The reduction in CRVE during NBR was highest on day 1 (DeltaCRVE = -7.9 mum; 95 CI: -13.3 to -2.5), and the maximum DeltaCRVE during HAMB (24.6 mum; 95% CI: 18.9-30.3) and HBR (15.2 mum; 95% CI: 9.8-20.5) was observed on days 10 and 3, respectively. Conclusions: **The diameters of retinal blood vessels exhibited a dynamic response to hypoxia and bed rest, such that retinal vasodilation was smaller during combined bed rest and hypoxia than during hypoxic exposure.**
(Rittweger et al., 2016) [7]	Bone losses are common as a consequence of unloading and also in patients with chronic obstructive pulmonary disease (COPD). Although hypoxia has been implicated as an important factor to drive bone loss, its interaction with unloading remains unresolved. The objective therefore was to assess whether human bone loss caused by unloading could be aggravated by chronic hypoxia. In a cross-over designed study, 14 healthy young men underwent 21-day interventions of bed rest in normoxia (NBR), bed rest in hypoxia (HBR), and hypoxic ambulatory confinement (HAmb). Hypoxic conditions were equivalent to 4000m altitude. **Bone metabolism (NTX, P1NP, sclerostin, DKK1) and phospho-calcic homeostasis (calcium and phosphate serum levels and urinary excretion, PTH) were assessed from regular blood samples and 24-hour urine collections, and tibia and femur bone mineral content was assessed by peripheral quantitative computed tomography (pQCT). Urinary NTX excretion increased (P<0.001) to a similar extent in NBR and HBR (P=0.69) and P1NP serum levels decreased (P=0.0035) with likewise no difference between NBR and HBR (P=0.88). Serum total calcium was increased during bed rest by 0.059 (day D05, SE 0.05mM) to 0.091mM (day D21, P<0.001), with no additional effect by hypoxia during bed rest (P=0.199). HAmb led, at least temporally, to increased total serum calcium, to reduced serum phosphate, and to reduced phosphate and calcium excretion. In conclusion, hypoxia did not aggravate bed rest-induced bone resorption, but led to changes in phospho-calcic homeostasis likely caused by hyperventilation.** Whether hyperventilation could have mitigated the effects of hypoxia in this study remains to be established.
(Rullman et al., 2016) [8]	The study concerns effects of 21 days of sustained bedrest and hypoxia, alone and in combination, on skeletal muscle microRNA (miRNA) expression. It is expected that astronauts undertaking long-duration missions will be exposed not only to microgravity but also to a hypoxic environment. The molecular machinery underlying microgravity-induced alterations in skeletal muscle structure and function is still largely unknown. One possible regulatory mechanism is altered expression of miRNAs, a group of noncoding RNAs which down-regulate many different target genes through increased degradation or translation of their messenger RNA. Thirteen healthy men underwent three 21-day interventions,

	interspersed by 4-month washout periods: horizontal bedrest in normoxia, bedrest in hypoxia, ambulation in hypoxia. The level of hypoxia corresponded to 4000 m altitude. miRNAs from v. lateralis muscle biopsies were analyzed using a microarray covering ≈4000 human miRNAs. **Sixteen mature miRNAs were up-regulated and three down-regulated after bedrest. The magnitudes of these changes were small and a large portion of the miRNAs affected by bedrest was also differentially expressed after washout periods. In fact, the number of differentially expressed probe sets over time was substantially larger than what could be detected after bedrest. Still, the majority of the miRNAs (let-7, miR-15, miR-25, miR-199, miR-133) that were differentially expressed following bedrest, belong to miRNA families previously reported in the context of muscle physiology, in particular to respond to changes in mechanical loading. Since only minor changes in miRNA expression could be detected after bedrest, our data indicate miRNA to play only a minor role in the substantial change in muscle phenotype seen with unloading.**
(Simpson et al., 2016) [9]	PlanHab is a planetary habitat simulation study. The atmosphere within future space habitats is anticipated to have reduced Po2, but information is scarce as to how physiological systems may respond to combined exposure to moderate hypoxia and reduced gravity. This study investigated, using a randomized-crossover design, how insulin sensitivity, glucose tolerance, and circulating lipids were affected by 16 days of horizontal bed rest in normobaric normoxia [NBR: FiO2 = 0.209; PiO2 = 133.1 (0.3) mmHg], horizontal bed rest in normobaric hypoxia [HBR: FiO2 = 0.141 (0.004); PiO2 = 90.0 (0.4) mmHg], and confinement in normobaric hypoxia combined with daily moderate intensity exercise (HAMB). A mixed-meal tolerance test, with arterialized-venous blood sampling, was performed in 11 healthy, nonobese men (25-45 yr) before (V1) and on the morning ofday 17of each intervention (V2). **Postprandial glucose and c-peptide response were increased at V2 of both bed rest interventions (P< 0.05 in each case), with c-peptide:insulin ratio higher at V2 in HAMB and HBR, both in the fed and fasted state (P< 0.005 in each case). Fasting total cholesterol was reduced at V2 in HAMB [-0.47 (0.36) mmol/l;P< 0.005] and HBR [-0.55 (0.41) mmol/l;P< 0.005]. Fasting HDL was lower at V2 in all interventions, with the reduction observed in HBR [-0.30 (0.21) mmol/l] greater than that measured in HAMB [-0.13 (0.14) mmol/l;P< 0.005] and NBR [-0.17 (0.15) mmol/l;P< 0.05]. Hypoxia did not alter the adverse effects of bed rest on insulin sensitivity and glucose tolerance but appeared to increase insulin clearance. The negative effect of bed rest on HDL was compounded in hypoxia, which may have implications for long-term health of those living in future space habitats.**

(Sket et al., 2017a) [1]	We explored the assembly of intestinal microbiota in healthy male participants during the run-in (5 day) and experimental phases [21-day normoxic bed rest (NBR), hypoxic bedrest (HBR)], and hypoxic ambulation (HAmb) in a strictly controlled laboratory environment, balanced fluid, and dietary intakes, controlled circadian rhythm, microbial ambiental burden, and 24/7 medical surveillance. The fraction of inspired O-2 (FiO2) and partial pressure of inspired O-2 (PiO2) were 0.209 and 133.1 +/- 0.3 mmHg for NBR and 0.141 +/- 0.004 and 90.0 +/- 0.4 mmHg for both hypoxic variants (HBR and HAmb; +/- 4,000m simulated altitude), respectively. A number of parameters linked to intestinal transit spanning Bristol Stool Scale, defecation rates, zonulin, a 1-antitrypsin, eosinophil derived neurotoxin, bile acids, reducing sugars, short chain fatty acids, total soluble organic carbon, water content, diet composition, and food intake weremeasured (167 variables). **The abundance, structure, and diversity of butyrate producing microbial community were assessed using the two primary bacterial butyrate synthesis pathways, butyryl-CoA: acetate CoA-transferase (but) and butyrate kinase (buk) genes. Inactivity negatively affected fecal consistency and in combination with hypoxia aggravated the state of gut inflammation ($p < 0.05$). In contrast, gut permeability, various metabolic markers, the structure, diversity, and abundance of butyrate producing microbial community were not significantly affected. Rearrangements in the butyrate producing microbial community structure were explained by experimental setup (13.4%), experimentally structured metabolites (12.8%), and gut metabolite-immunological markers (11.9%), with 61.9% remaining unexplained. Many of the measured parameters were found to be correlated and were hence omitted from further analyses. The observed progressive increase in two immunological intestinal markers suggested that the transition fromhealthy physiological state toward the developed symptoms of low magnitude obesity-related syndromes was primarily driven by the onset of inactivity (lack of exercise in NBR) that were exacerbated by systemic hypoxia (HBR) and significantly alleviated by exercise, despite hypoxia (HAmb). Butyrate producing community in colon exhibited apparent resilience toward short-termmodifications in host exercise or hypoxia. Progressive constipation (decreased intestinal motility) and increased local inflammation marker suggest that changes in microbial colonization and metabolism were taking place at the location of small intestine.**
(Sket et al., 2017b) [10]	We explored the assembly of intestinal microbiota in healthy male participants during the randomized crossover design of run-in (5 day) and experimental phases (21-day normoxic bed rest (NBR), hypoxic bed rest (HBR) and hypoxic ambulation (HAmb) in a strictly controlled laboratory environment, with balanced fluid and dietary intakes, controlled circadian rhythm, microbial ambiental burden and 24/7 medical surveillance. The fraction of inspired O-2 (FiO2) and partial pressure of inspired O-2 (PiO2) were 0.209 and 133.1 +/- 0.3 mmHg for NBR and 0.141 +/- 0.004 and 90.0 +/- 0.4 mmHg for both hypoxic variants (HBR and HAmb; similar to 4000 m simulated altitude), respectively. A number of parameters linked to intestinal environment such as defecation frequency, intestinal electrical conductivity (IEC), sterol and polyphenol content and diversity, indole, aromaticity and spectral characteristics of dissolved organic matter (DOM) were measured (64 variables). **The structure and diversity of bacterial microbial community was assessed using 16S rRNA amplicon sequencing. Inactivity negatively affected frequency of defecation and in combination with hypoxia increased IEC ($p < 0.05$). In contrast, sterol and polyphenol diversity and content, various characteristics of DOM and aromatic compounds, the structure and diversity of bacterial microbial community were not significantly affected over time. A new in-house PlanHab database was established to integrate all measured variables on host physiology, diet, experiment, immune and metabolic markers ($n = 231$). The observed progressive decrease in defecation frequency and concomitant increase in IEC suggested that the transition from healthy physiological state towards the developed symptoms of low magnitude obesity-related syndromes was dose dependent on the extent of time spent in inactivity and preceded or took place in absence of significant rearrangements in bacterial microbial community. Species B. thetaiotamicron, B. fragilis, B. dorei and other Bacteroides with reported relevance for dysbiotic medical conditions were significantly enriched in HBR, characterized with most severe inflammation symptoms, indicating a shift towards host mucin degradation and proinflammatory immune crosstalk.**
(Morrison et al., 2017) [11]	Objective: Despite over 50 years of research on the physiological effects of sustained bed rest, data characterizing its effects on sleep macrostructure and breathing stability in humans

	are scarce. This study was conducted to determine the effects of continuous exposure to hypoxia and sustained best rest, both individually and combined, on nocturnal sleep and breathing stability.Methods: Eleven participants completed three randomized, counter-balanced, 21-days trials of: (1) normoxic bed rest (NBR, PIO2 = 133.1 ± 0.3), (2) hypoxic ambulatory confinement (HAMB, PIO2 = 90.0 ± 0.4) and (3) hypoxic bed rest (HBR, PIO2 = 90.0 ± 0.4; ~4,000 m equivalent altitude). Full objective polysomnography was performed at baseline, on Night 1 and Night 21 in each condition.Results: **In NBR Night 1, more time was spent in light sleep (10 ± 2%) compared to baseline (8 ± 2%; p = 0.028); Slow-wave sleep (SWS) was reduced from baseline in the hypoxic-only trial by 18% (HAMB Night 21, p = 0.028) and further reduced by 33% (HBR Night 1, p = 0.010), and 36% (HBR Night 21, p = 0.008) when combined with bed rest. The apnea-hypopnea index doubled from Night 1 to Night 21 in HBR (32–62 events·h−1) and HAMB (31–59 events·h−1; p = 0.002). Those who experienced greatest breathing instability from Night 1 to Night 21 (NBR) were correlated to unchanged or higher (+1%) night SpO2 concentrations (R2 = 0.471, p = 0.020).Conclusion: Bed rest negatively affects sleep macrostructure, increases the apnea-hypopnea index, and worsens breathing stability, each independently exacerbated by continuous exposure to hypoxia.**
(Strewe et al., 2017) [12]	Immobilization and hypoxemia are conditions often seen in patients suffering from severe heart insufficiency or primary pulmonary diseases (e.g. fibrosis, emphysema). In future planned long-duration and exploration class space missions (including habitats on the moon and Mars), healthy individuals will encounter such a combination of reduced physical activity and oxygen tension by way of technical reasons and the reduced gravitational forces. These overall unconventional extraterrestrial conditions can result in yet unknown consequences for the regulation of stress-permissive, psycho-neuroendocrine responses, which warrant appropriate measures in order to mitigate foreseeable risks. The Planetary Habitat Simulation Study (PlanHab) investigated these two space-related conditions: bed rest as model of reduced gravity and normobaric hypoxia, with the aim of examining their influence on psycho-neuroendocrine responses. We hypothesized that both conditions independently increase measures of psychological stress and enhance neuroendocrine markers of stress, and that these effects would be exacerbated by combined treatment. The cross-over study composed of three interventions (NBR, normobaric normoxic horizontal bed rest; HBR, normobaric hypoxic horizontal bed rest; HAMB, normobaric hypoxic ambulatory confinement) with 14 male subjects during three sequential campaigns separated by 4 months. The psychological state was determined through three questionnaires and principal neuroendocrine responses were evaluated by measuring cortisol in saliva, catecholamine in urine, and endocannabinoids in blood. **The results revealed no effects after 3 weeks of normobaric hypoxia on psycho-neuroendocrine responses. Conversely, bed rest induced neuroendocrine alterations that were not influenced by hypoxia.**
(Debevec et al., 2018) [13]	Poor musculoskeletal state is commonly observed in numerous clinical populations such as chronic obstructive pulmonary disease (COPD) and heart failure patients. It, however, remains unresolved whether systemic hypoxemia, typically associated with such clinical conditions, directly contributes to muscle deterioration. We aimed to experimentally elucidate the effects of systemic environmental hypoxia upon inactivity-related muscle wasting. For this purpose, fourteen healthy, male participants underwent three 21-day long interventions in a randomized, cross-over designed manner: (i) bed rest in normoxia (NBR; PiO2 = 133.1 +/- 0.3 mmHg), (ii) bed rest in normobaric hypoxia (HBR; PiO2 = 90.0 +/- 0.4 mmHg) and ambulatory confinement in normobaric hypoxia (HAmb; PiO2 = 90.0 +/- 0.4 mmHg). Peripheral quantitative computed tomography and vastus lateralis muscle biopsies were performed before and after the interventions to obtain thigh and calf muscle cross-sectional areas and muscle fiber phenotype changes, respectively. **A significant reduction of thigh muscle size following NBR (-6.9%, SE 0.8%; P < 0.001) was further aggravated following HBR (-9.7%, SE 1.2%; P = 0.027). Bed rest-induced muscle wasting in the calf was, by contrast, not exacerbated by hypoxic conditions (P = 0.47). Reductions in both thigh (-2.7%, SE 1.1%, P = 0.017) and calf (-3.3%, SE 0.7%, P < 0.001) muscle size were noted following HAmb. A significant and comparable increase in type 2x fiber percentage of the vastus lateralis muscle was noted following both bed rest interventions (NBR = +3.1%, SE 2.6%, HBR = +3.9%, SE 2.7%, P < 0.05). Collectively, these data indicate that hypoxia can exacerbate inactivity-related muscle wasting in healthy active participants and moreover suggest that the combination of both, hypoxemia and lack of activity, as seen in COPD patients, might be particularly harmful for muscle tissue.**

(Sket et al., 2018) [14]	We explored the metagenomic, metabolomic and trace metal makeup of intestinal microbiota and environment in healthy male participants during the run-in (5 day) and the following three 21-day interventions: normoxic bedrest (NBR), hypoxic bedrest (HBR) and hypoxic ambulation (HAmb) which were carried out within a controlled laboratory environment (circadian rhythm, fluid and dietary intakes, microbial bioburden, oxygen level, exercise). The fraction of inspired O-2 (FiO(2)) and partial pressure of inspired O-2 (PiO2) were 0.209 and 133.1 +/- 0.3 mmHg for the NBR and 0.141 +/- 0.004 and 90.0 +/- 0.4 mmHg (similar to 4,000m simulated altitude) for HBR and HAmb interventions, respectively. Shotgun metagenomes were analyzed at various taxonomic and functional levels, H-1- and C-13 - metabolomes were processed using standard quantitative and human expert approaches, whereas metals were assessed using X-ray fluorescence spectrometry**. Inactivity and hypoxia resulted in a significant increase in the genus Bacteroides in HBR, in genes coding for proteins involved in iron acquisition and metabolism, cell wall, capsule, virulence, defense and mucin degradation, such as beta-galactosidase (EC3.2.1.23), alpha-L-fucosidase (EC3.2.1.51), Sialidase (EC3.2.1.18), and alpha-N-acetylglucosaminidase (EC3.2.1.50). In contrast, the microbial metabolomes, intestinal element and metal profiles, the diversity of bacterial, archaeal and fungal microbial communities were not significantly affected.** The observed progressive decrease in defecation frequency and concomitant increase in the electrical conductivity (EC) preceded or took place in absence of significant changes at the taxonomic, functional gene, metabolome and intestinal metal profile levels. **The fact that the genus Bacteroides and proteins involved in iron acquisition and metabolism, cell wall, capsule, virulence and mucin degradation were enriched at the end of HBR suggest that both constipation and EC decreased intestinal metal availability leading to modified expression of co-regulated genes in Bacteroides genomes.** Bayesian network analysis was used to derive the first hierarchical model of initial inactivity mediated deconditioning steps over time. **The PlanHab wash-out period corresponded to a profound life-style change (i.e., reintroduction of exercise) that resulted in stepwise amelioration of the negative physiological symptoms, indicating that exercise apparently prevented the crosstalk between the microbial physiology, mucin degradation and proinflammatory immune activities in the host.**
(Rullman et al., 2018) [15]	Our understanding of skeletal muscle structural and functional alterations during unloading has increased in recent decades, yet the molecular mechanisms underpinning these changes have only started to be unraveled. The purpose of the current investigation was to assess changes in skeletal muscle gene expression after 21 days of bed rest, with a particular focus on predicting upstream regulators of muscle disuse. Additionally, the association between differential microRNA expression and the transcriptome signature of bed rest were investigated. mRNAs from musculus vastus lateralis biopsies obtained from 12 men before and after the bed rest were analyzed using a microarray. There were 54 significantly upregulated probesets after bed rest, whereas 103 probesets were downregulated (false discovery rate 10%; fold-change cutoff >/=1.5). **Among the upregulated genes, transcripts related to denervation-induced alterations in skeletal muscle were identified, e.g., acetylcholine receptor subunit delta and perinatal myosin. The most downregulated transcripts were functionally enriched for mitochondrial genes and genes involved in mitochondrial biogenesis, followed by a large number of contractile fiber components.** Upstream regulator analysis identified a robust inhibition of the myocyte enhancer factor-2 (MEF2) family, in particular MEF2C, which was suggested to act upstream of several key downregulated genes, most notably peroxisome proliferator-activated receptor gamma coactivator 1-alpha (PGC-1alpha)/peroxisome proliferator-activated receptors (PPARs) and CRSP3. **Only a few microRNAs were identified as playing a role in the overall transcriptome picture induced by sustained bed rest. Our results suggest that the MEF2 family is a key regulator underlying the transcriptional signature of bed rest and, hence, ultimately also skeletal muscle alterations induced by systemic unloading in humans.**
(Salvadego et al., 2018) [16]	ABSTRACT: Skeletal muscle oxidative function was evaluated in 11 healthy males (mean +/- SD age 27 +/- 5 years) prior to (baseline data collection, BDC) and following a 21 day horizontal bed rest (BR), carried out in normoxia (PIO2 = 133 mmHg; N-BR) and hypoxia (PIO2 = 90 mmHg; H-BR). H-BR was aimed at simulating reduced gravity habitats. The effects of a 21 day hypoxic ambulatory confinement (PIO2 = 90 mmHg; H-AMB) were also assessed. Pulmonary O2 uptake (VO2), vastus lateralis fractional O2 extraction (changes in deoxygenated haemoglobin + myoglobin concentration, Delta[deoxy(Hb + Mb)]; near-

	infrared spectroscopy) and femoral artery blood flow (ultrasound Doppler) were evaluated during incremental one-leg knee-extension exercise (reduced constraints to cardiovascular O2 delivery) carried out to voluntary exhaustion in a normoxic environment. Mitochondrial respiration was evaluated ex vivo by high-resolution respirometry in permeabilized vastus lateralis fibres. **VO2peak decreased (P < 0.05) after N-BR (0.98 +/- 0.13 L min(-1)) and H-BR (0.96 +/- 0.17 L min(-1)) vs. BDC (1.05 +/- 0.14 L min(-1)). In the presence of a decreased (by approximately 6-8%) thigh muscle volume, VO2peak normalized per unit of muscle mass was not affected by both interventions. Delta[deoxy(Hb + Mb)]peak decreased (P < 0.05) after N-BR (65 +/- 13% of limb ischaemia) and H-BR (62 +/- 12%) vs. BDC (73 +/- 13%). H-AMB did not alter VO2peak or Delta[deoxy(Hb + Mb)]peak . An overshoot of Delta[deoxy(Hb + Mb)] was evident during the first minute of unloaded exercise after N-BR and H-BR. Arterial blood flow to the lower limb during both unloaded and peak knee extension was not affected by any intervention. Maximal ADP-stimulated mitochondrial respiration decreased (P < 0.05) after all interventions vs. control. In 21 day N-BR, a significant impairment of oxidative metabolism occurred downstream of cardiovascular O2 delivery, affecting both mitochondrial respiration and presumably the intramuscular matching between O2 supply and utilization.** Superposition of H on BR did not worsen the impairment induced by BR alone.
(Stavrou et al., 2018a) [17]	Hypoxia and confinement have both been shown to influence emotional state. It is envisaged that the inhabitants of future planetary habitats will be exposed to concomitant confinement, reduced gravity and hypoxia. We examined the independent and combined effects of a 21-day inactivity/unloading and normobaric hypoxia under confined conditions on various psychological factors. Eleven healthy men participated in three 21-day experimental campaigns designed in a cross-over manner: (1) Normobaric hypoxic ambulatory confinement, (2) Normobaric hypoxic bed rest and (3) Normobaric normoxic bed rest. The Profile of Mood States, and the Positive and Negative Affect Schedule were employed to assess the participants' psychological responses before (Pre), during (Day 7, Day 14, and Day 21) and after (Post) the confinements. **The most negative psychological profile appeared on days 14 and 21 of the hypoxic bed rest campaign. A significant increase in depression, tension, and confusion was noted on days 14 and 21 of the hypoxic bed rest condition. Concomitantly, a decrease, albeit not statistically significant, in positive psychological responses was observed. The psychological profile returned to the initial level at Post following all confinements. These data suggest that the combined effect of hypoxia and bed rest induced the most negative effects on an individual's mood. However, significant intra- and inter-individual differences in psychological responses were noted and should be taken into consideration.**
(Stavrou et al., 2018b) [18]	Previous research, although limited, suggests that both hypoxia and bed rest influence psychological responses by exaggerating negative psychological responses and attenuating positive emotions. The present study investigated the effect of a 21-day prolonged exposure to normobaric hypoxia and bed rest on affective responses and fatigue. Eleven healthy participants underwent three 21-day interventions using a cross-over design: (1) normobaric hypoxic ambulatory confinement (HAMB), (2) normobaric hypoxic bed rest (HBR) and (3) normoxic bed rest (NBR). Affective and fatigue responses were investigated using the Activation Deactivation Adjective Check List, and the Multidimensional Fatigue Inventory, which were completed before (Pre), during (Day 7, Day 14, and Day 21) and after (Post) the interventions. **The most negative psychological profile appeared during the HBR intervention. Specifically, tiredness, tension, general and physical fatigue significantly increased on days 7, 14, and 21, as well as at Post. After the HBR intervention, general and physical fatigue remained higher compared to Pre values. Additionally, a deterioration of psychological responses was also noted following HAMB and NBR. In particular, both hypoxia and BR per se induced subjective fatigue and negative affective responses. BR seems to exert a moderate negative effect on the sensation of fatigue, whereas exercise attenuates the negative effects of hypoxia as noted during the HAMB condition. In conclusion, our data suggest that the addition of hypoxia to bed rest-induced inactivity significantly worsens affective responses and feeling of fatigue.**
(Strewe et al., 2018) [19]	Adenosine plays a role in the energy supply of cells and provokes differential, hormone-like functions in circulating cells and various tissues. Its release is importantly regulated by oxygen tension. This renders adenosine and its kinetics interesting to investigate in humans subjected to low oxygen conditions. Especially for space exploration scenarios, hypoxic conditions - together with reduced gravity - represent two foreseen living conditions when

	planning manned long-duration space missions or planetary habitats. The PlanHab study investigated microgravity through inactivity in bed rest and normobaric hypoxia to examine their independent or combined effect on adenosine and its kinetics. Healthy male subjects (n = 14) completed three 21-day interventions: hypoxic bed rest (HBR); hypoxic ambulatory confinement (HAMB); normoxic bed rest (NBR). The interventions were separated by 4 months. **Our hypothesis of a hypoxia-triggered increase in adenosine was confirmed in HAMB but unexpectedly also in NBR. However, the highest adenosine levels were noted following HBR. Furthermore, the percentage of hemolysis was elevated in HBR whereas endothelial integrity markers stayed low in all three interventions. In summary, these data suggest that neocytolysis accounts for these effects while we could reduce evidence for microcirculatory changes.**
(Sarabon et al., 2018) [20]	Prolonged inactivity, such as bed rest induces several detrimental changes within a short timeframe. Impaired postural balance and responses of trunk muscles to (un)expected perturbations were both shown to be impaired after bed rest. Certain populations (e.g., astronauts) are exposed to hypoxic environment in addition to inactivity, similar to bed rest. While the isolated negative effects of hypoxia on postural balance have been observed before, no study to date has examined the combined effects of hypoxia and bed rest on postural balance or trunk muscle responses. In this study, we examined the effects of 21-day exposure to three conditions: (i) bed rest in hypoxic environment (HBR), (ii) bed rest in normoxic environment (NBR), and (iii) ambulatory hypoxic environment (HAMB). Fourteen healthy male subjects crossed over between conditions in a randomized order, with a 4-month break between conditions to ensure full recovery. Most body sway parameters indicated a similar deterioration of postural balance following both HBR and NBR. **Similarly, both anticipatory and reactive responses of the trunk muscles (m. erector spinae and m. multifidus) were impaired after HBR and NBR to a similar degree and mostly unchanged after HAMB. Certain body sway parameters were impaired after HAMB, confirming that hypoxia alone can undermine postural balance. On the other hand, some trunk responses were improved after HAMB. In conclusion, the results of our study confirmed previous findings on negative effects of bed rest, but showed little or no additional effect of hypoxia during bed rest. Physical activity during bed rest is encouraged to preserve neuromuscular functions of the trunk.** While the HBR condition in our study resembled conditions during space missions, our results could be relevant to other populations, such as patients with pulmonary diseases exposed to bed rest.
(Ciuha et al., 2020) [21]	Seasonal variations in day length and light intensity can affect the circadian rhythm as well as some characteristics of temperature regulation. We investigated characteristics of autonomic (ATR), behavioural (BTR) and nocturnal (NTR) temperature regulation during spring and autumn. Eleven participants underwent experiments in both seasons. To assess ATR, participants performed a 30-min bout of submaximal upright exercise on a cycle ergometer, followed by 100 min of water immersion (28 °C). Thresholds for the onset of shivering and sweating and vasomotor response were measured. BTR was assessed using a water-perfused suit, with participants regulating the water-perfused suit temperature (Twps) within a range, considered as thermally comfortable. The Twps changed in a saw-tooth manner from 10 to 50 °C; by depressing a switch, the direction of the Twps changed, and this limit defined the thermal comfort zone (TCZ) for each participant. A 24-h proximal (calf)–distal (toe) skin temperature gradient (ΔTc-t) was measured to assess NTR. Initiation of vasomotor tone, shivering and sweating was similar between trials. Width of the TCZ was 8.1 °C in spring and 8.6 °C in autumn ($p = 0.1$), with similar upper and lower regulated temperatures. ΔTc-t exhibited a typical circadian rhythm with no difference between seasons. **Minor changes in skin temperature and oxygen consumption ($p < 0.05$) between the seasons may indicate a degree of seasonal adaptation over the course of winter and summer, which persisted in spring and autumn**. Other factors, such as country, race, sex and age could however modify the outcome of the study.

(Šket et al., 2020) [2]	We explored the metabolic makeup of urine in prescreened healthy male participants within the PlanHab experiment. The run-in (5 day) and the following three 21-day interventions [normoxic bedrest (NBR), hypoxic bedrest (HBR), and hypoxic ambulation (HAmb)] were executed in a crossover manner within a controlled laboratory setup (medical oversight, fluid and dietary intakes, microbial bioburden, circadian rhythm, and oxygen level). The inspired O2 (FiO2) fraction next to inspired O2 (PiO2) partial pressure were 0.209 and 133.1 ± 0.3 mmHg for the NBR variant in contrast to 0.141 ± 0.004 and 90.0 ± 0.4 mmHg (approx. 4,000 m of simulated altitude) for HBR and HAmb interventions, respectively. 1H-NMR metabolomes were processed using standard quantitative approaches. A consensus of ensemble of multivariate analyses showed that the metabolic makeup at the start of the experiment and at HAmb endpoint differed significantly from the NBR and HBR endpoints. Inactivity alone or combined with hypoxia resulted in a significant reduction of metabolic diversity and increasing number of affected metabolic pathways. Sliding window analysis (3 + 1) unraveled that metabolic changes in the NBR lagged behind those observed in the HBR. **These results show that the negative effects of cessation of activity on systemic metabolism are further aggravated by additional hypoxia. The PlanHab HAmb variant that enabled ambulation, maintained vertical posture, and controlled but limited activity levels apparently prevented the development of negative physiological symptoms such as insulin resistance, low-level systemic inflammation, constipation, and depression. This indicates that exercise apparently prevented the negative spiral between the host's metabolism, intestinal environment, microbiome physiology, and proinflammatory immune activities in the host.**

Instructions for running a model on a local machine

JADBIO allows the user to download a model and run it on a local machine.

To run our model locally, the user must meet the following requirements:

1. Java SE Development Kit version 15 (https://www.oracle.com/java/technologies/javase-jdk15-downloads.html)

2. the Java executor (contained in model.zip - filename: jadbio-1.1.182-model-exe.jar)

3. model (contained in model.zip - filename: jadbio-1.1.182-model-MP.bin)

After installing Java SE JDK, model.zip must be saved somewhere on the local machine. After saving model.zip, provided by the authors, the folder must be extracted (e.g. with WinZip, 7zip). The model must be executed with the command prompt (cmd) (Fig. 1, 2).

Step 1

Using the `cd path` command (Fig. 1), the user navigates to the same directory (e.g. Folder) that contains the model executor (.jar) and the model (.bin).

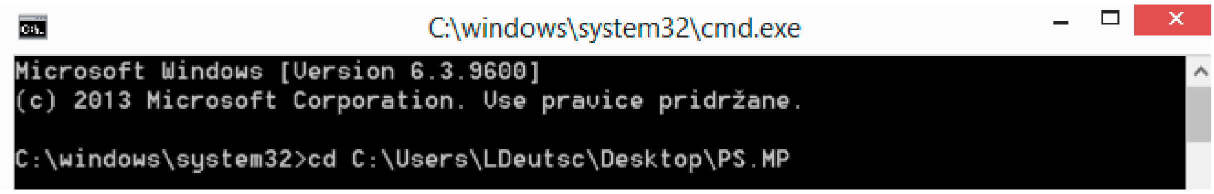

Figure I1. First command to navigate to the folder containing the model. In this case, we used the `cd C:\Users\LDeutsc\Desktop\PS.MP` command because Executor and Model were in the PS.MP folder on the desktop.

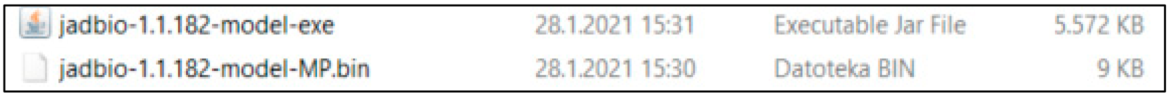

Figure I2. The two files needed for the overview of the model. Both files are contained in the model.zip folder.

Step 2

The next step is to preview the model using the following command:

```
Java --enable-preview -jar jadbio-1.1.182-model-exe.jar -n jadbio-1.1.182-model-MP.bin
```

This allows the user to get an overview of the model, key features and information about the analysis (which algorithm was used, version of JADBIO and other information about the model) (Fig. 3).

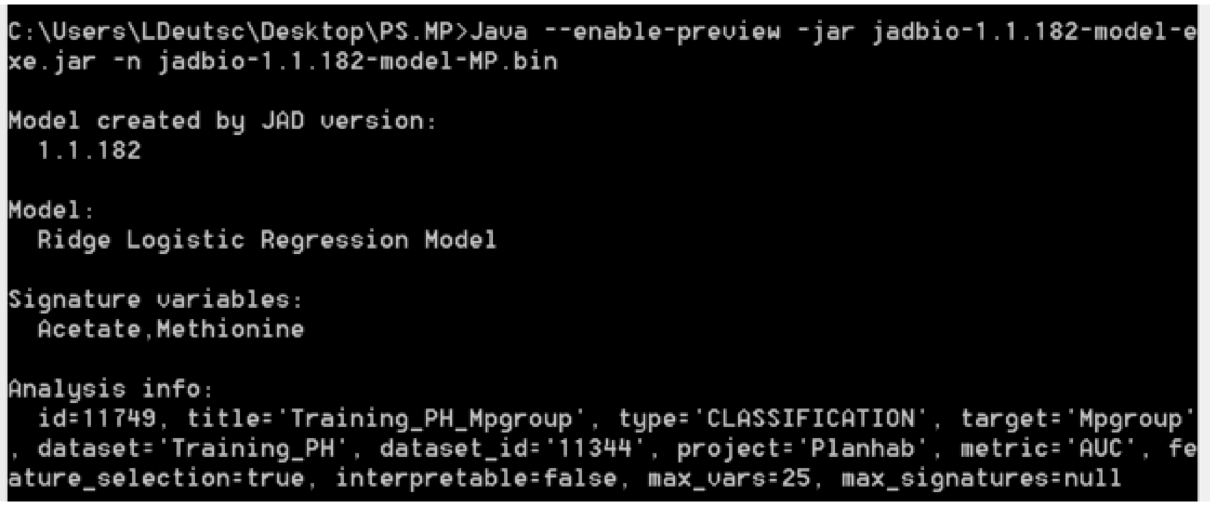

Figure I3. Overview of the model.

Step 3

To test the model, the user must prepare data. For the model to work properly, the concentrations must be in µM. The user needs to prepare the dataset as shown in Figure 4 and save it in a .csv document (comma separated values). After preparation, the prepared dataset must be saved in the same directory as the model executor and the model itself.

	A	B	C	D
1	Sample	Mpgroup	Acetate	Methionine
2	MP1	1	36.6	0
3	MP1	1	50.8	0
4	MP1	1	37.7	0.8
5	MP1	1	34.1	0
6	MP1	1	54.2	0.8
7	MP2	2	16.4	0.4
8	MP2	2	0	0
9	MP2	2	40.1	2.6
10	MP3	3	12	0
11	MP3	3	9	0
12	MP3	3	16	0

Figure I4. Prepared dataset.

Step 4

After saving the dataset, the user must use the next command in the terminal:

```
java --enable-preview -jar jadbio-1.1.182-model-exe.jar -m jadbio-1.1.182-model-MP.bin -i MPtest.csv -o MPtest-output.csv
```

This command runs the model on test data (MPtest.csv in our case) and creates a new dataset with predictions (MPtest-output.csv) (Fig. 5).

```
C:\Users\LDeutsc\Desktop\PS.MP>java --enable-preview -jar jadbio-1.1.182-model-e
xe.jar -m jadbio-1.1.182-model-MP.bin -i MPtest.csv -o MPtest-output.csv
Successfully loaded model from jadbio-1.1.182-model-MP.bin
Successfully loaded input dataset from MPtest.csv
Successfully wrote predictions to MPtest-output.csv
```

Figure I5. Executing the model and creating the output .csv file with predictions in the same directory.

Step 5

After model execution, the user can check the calculated predictions by opening the .csv file directly by clicking on the created .csv file and opening it in any data analysis program (Excel, Past, R ...). As shown in Fig. 6 the model classifies the data between different classes (MP1, MP2 and MP3). The first column is the same as in the test data created by the user. For issues with classification of MP2 class please see also Figure 2 in the original publication (Deutsch and Stres, 2021)

1	Sample name,Prob (class = 1),Prob (class = 2),Prob (class = 3)
2	MP1,0.8533318778399783,0.07711875023190991,0.06954937192811184
3	MP1,0.9725501497380422,0.017560742309497408,0.009889107952460396
4	MP1,0.810747769551975,0.0963415720927212,0.0929106509520813 8
5	MP1,0.8080319522931302,0.09696306009973585,0.0950049876071340 1
6	MP1,0.9722931805279149,0.0177839877127985,0.00992283175928651 7
7	MP2,0.24310085900279949,0.2650320127877801,0.4918671282094205
8	MP2,0.04139610883000549,0.23752489793050943,0.7210789932394851
9	MP2,0.67966296916884,0.1508026695044212,0.16953436132673877
0	MP3,0.18276826394176032,0.268904910065478 7,0.548326825992761
1	MP3,0.12945634184681387,0.26766044453777565,0.6028832136154105
2	MP3,0.27747514368328263,0.2593610799132837,0.46316377640343365
3	

Figure I6. The newly created .csv file with predictions calculated from test data.

PLANHAB REFERENECES

1. Sket, R.; Treichel, N.; Debevec, T.; Eiken, O.; Mekjavic, I.; Schloter, M.; Vital, M.; Chandler, J.; Tiedje, J.M.; Murovec, B.; et al. Hypoxia and Inactivity Related Physiological Changes (Constipation, Inflammation) Are Not Reflected at the Level of Gut Metabolites and Butyrate Producing Microbial Community: The PlanHab Study. *Frontiers in Physiology* **2017**, *8*, doi:10.3389/fphys.2017.00250.
2. Šket, R.; Deutsch, L.; Prevoršek, Z.; Mekjavić, I.B.; Plavec, J.; Rittweger, J.; Debevec, T.; Eiken, O.; Stres, B. Systems View of Deconditioning During Spaceflight Simulation in the PlanHab Project: The Departure of Urine 1 H-NMR Metabolomes From Healthy State in Young Males Subjected to Bedrest Inactivity and Hypoxia. *Frontiers in physiology* **2020**, *11*, doi:10.3389/fphys.2020.532271.
3. Debevec, T.; Bali, T.C.; Simpson, E.J.; Macdonald, I.A.; Eiken, O.; Mekjavic, I.B. Separate and combined effects of 21-day bed rest and hypoxic confinement on body composition. *Eur J Appl Physiol* **2014**, *114*, 2411-2425, doi:10.1007/s00421-014-2963-1.

4. Debevec, T.; Simpson, E.J.; Mekjavic, I.B.; Eiken, O.; Macdonald, I.A. Effects of prolonged hypoxia and bed rest on appetite and appetite-related hormones. *Appetite* **2016**, *107*, 28-37, doi:10.1016/j.appet.2016.07.005.
5. Keramidas, M.E.; Kolegard, R.; Mekjavic, I.B.; Eiken, O. PlanHab: hypoxia exaggerates the bed-rest-induced reduction in peak oxygen uptake during upright cycle ergometry. *Am J Physiol Heart Circ Physiol* **2016**, *311*, H453-464, doi:10.1152/ajpheart.00304.2016.
6. Louwies, T.; Jaki Mekjavic, P.; Cox, B.; Eiken, O.; Mekjavic, I.B.; Kounalakis, S.; De Boever, P. Separate and Combined Effects of Hypoxia and Horizontal Bed Rest on Retinal Blood Vessel Diameters. *Invest Ophthalmol Vis Sci* **2016**, *57*, 4927-4932, doi:10.1167/iovs.16-19968.
7. Rittweger, J.; Debevec, T.; Frings-Meuthen, P.; Lau, P.; Mittag, U.; Ganse, B.; Ferstl, P.G.; Simpson, E.J.; Macdonald, I.A.; Eiken, O.; et al. On the combined effects of normobaric hypoxia and bed rest upon bone and mineral metabolism: Results from the PlanHab study. *Bone* **2016**, *91*, 130-138, doi:10.1016/j.bone.2016.07.013.
8. Rullman, E.; Mekjavic, I.B.; Fischer, H.; Eiken, O. PlanHab (Planetary Habitat Simulation): the combined and separate effects of 21 days bed rest and hypoxic confinement on human skeletal muscle miRNA expression. In *Physiol Rep*; 2016; Volume 4.
9. Simpson, E.J.; Debevec, T.; Eiken, O.; Mekjavic, I.; Macdonald, I.A. PlanHab: the combined and separate effects of 16 days of bed rest and normobaric hypoxic confinement on circulating lipids and indices of insulin sensitivity in healthy men. *J Appl Physiol (1985)* **2016**, *120*, 947-955, doi:10.1152/japplphysiol.00897.2015.
10. Sket, R.; Treichel, N.; Kublik, S.; Debevec, T.; Eiken, O.; Mekjavic, I.; Schloter, M.; Vital, M.; Chandler, J.; Tiedje, J.M.; et al. Hypoxia and inactivity related physiological changes precede or take place in absence of significant rearrangements in bacterial community structure: The PlanHab randomized trial pilot study. *Plos One* **2017**, *12*, doi:10.1371/journal.pone.0188556.
11. Morrison, S.A.; Mirnik, D.; Korsic, S.; Eiken, O.; Mekjavic, I.B.; Dolenc-Groselj, L. Bed Rest and Hypoxic Exposure Affect Sleep Architecture and Breathing Stability. *Front Physiol* **2017**, *8*, doi:10.3389/fphys.2017.00410.
12. Strewe, C.; Zeller, R.; Feuerecker, M.; Hoerl, M.; Kumprej, I.; Crispin, A.; Johannes, B.; Debevec, T.; Mekjavic, I.; Schelling, G.; et al. PlanHab study: assessment of psycho-neuroendocrine function in male subjects during 21 d of normobaric hypoxia and bed rest. *Stress* **2017**, *20*, 131-139, doi:10.1080/10253890.2017.1292246.
13. Debevec, T.; Ganse, B.; Mittag, U.; Eiken, O.; Mekjavic, I.B.; Rittweger, J. Hypoxia Aggravates Inactivity-Related Muscle Wasting. *Front Physiol* **2018**, *9*, 494, doi:10.3389/fphys.2018.00494.
14. Sket, R.; Debevec, T.; Kublik, S.; Schloter, M.; Schoeller, A.; Murovec, B.; Mikus, K.V.; Makuc, D.; Pecnik, K.; Plavec, J.; et al. Intestinal Metagenomes and Metabolomes in Healthy Young Males: Inactivity and Hypoxia Generated Negative Physiological Symptoms Precede Microbial Dysbiosis. *Frontiers in Physiology* **2018**, *9*, doi:10.3389/fphys.2018.00198.
15. Rullman, E.; Fernandez-Gonzalo, R.; Mekjavic, I.B.; Gustafsson, T.; Eiken, O. MEF2 as upstream regulator of the transcriptome signature in human skeletal muscle during unloading. *Am J Physiol Regul Integr Comp Physiol* **2018**, *315*, R799-r809, doi:10.1152/ajpregu.00452.2017.
16. Salvadego, D.; Keramidas, M.E.; Kolegard, R.; Brocca, L.; Lazzer, S.; Mavelli, I.; Rittweger, J.; Eiken, O.; Mekjavic, I.B.; Grassi, B. PlanHab(*) : hypoxia does not worsen the impairment of skeletal muscle oxidative function induced by bed rest alone. *J Physiol* **2018**, *596*, 3341-3355, doi:10.1113/jp275605.
17. Stavrou, N.A.M.; Debevec, T.; Eiken, O.; Mekjavic, I.B. Hypoxia Exacerbates Negative Emotional State during Inactivity: The Effect of 21 Days Hypoxic Bed Rest and Confinement. *Front Physiol* **2018**, *9*, 26, doi:10.3389/fphys.2018.00026.
18. Stavrou, N.A.M.; Debevec, T.; Eiken, O.; Mekjavic, I.B. Hypoxia Worsens Affective Responses and Feeling of Fatigue During Prolonged Bed Rest. *Front Psychol* **2018**, *9*, 362, doi:10.3389/fpsyg.2018.00362.

19. Strewe, C.; Zeller, R.; Feuerecker, M.; Hoerl, M.; Matzel, S.; Kumprej, I.; Crispin, A.; Johannes, B.; Debevec, T.; Mekjavic, I.B.; et al. PlanHab Study: Consequences of combined normobaric hypoxia and bed rest on adenosine kinetics. *Sci Rep* **2018**, *8*, 1762, doi:10.1038/s41598-018-20045-5.
20. Sarabon, N.; Mekjavic, I.B.; Eiken, O.; Babic, J. The Effect of Bed Rest and Hypoxic Environment on Postural Balance and Trunk Automatic (Re)Actions in Young Healthy Males. *Front Physiol* **2018**, *9*, 27, doi:10.3389/fphys.2018.00027.
21. Ciuha, U.; Kounalakis, S.; McDonnell, A.C.; I.B., M. Seasonal variation of temperature regulation: do thermoregulatory responses “spring” forward and “fall” back? *International Journal of Biometeorology* **2020**, 1-11, doi:10.1007/s00484-020-01898-w.
